# Mode‐Resolved Phonon Dynamics Under Chemical Pressure in SnTe Thermoelectrics

**DOI:** 10.1002/advs.78136

**Published:** 2026-10-06

**Authors:** Zhihao Li, Jianhong Hu, Panpan Peng, Peng Cao, Hui Zhang, Yujie Zong, Yanhui Wu, Hongchao Wang

**Affiliations:** ^1^ School of Physics Shandong University Jinan People's Republic of China

**Keywords:** chemical pressure, phonon dynamics, SnTe thermoelectrics, spectroscopy

## Abstract

Chemical pressure is used to tune lattice thermal conductivity (*κ*_l_) in thermoelectric materials, yet its effect on phonon transport remains uncovered. Here, Sn_0.95_M_0.05_Te (M = Sn, Mn, Ge, Sm, and Pb) is used to investigate how substitution‐induced chemical pressure modifies phonon dynamics in SnTe. Mapping refined lattice parameters onto an equivalent pressure scale shows that Sn_0.95_M_0.05_Te deviates from the elasticity‐based *κ*_l_ trend defined by physical pressure, indicating a nonuniform influence of chemical pressure on different phonon modes. Terahertz time‐domain spectroscopy, Raman spectroscopy, and Brillouin spectroscopy resolve the Γ‐point optical mode, L‐point optical modes, two‐phonon Raman features, and long‐wavelength acoustic phonons. These measurements, combined with first‐principles lattice dynamics and spectral energy density analysis, show that chemical pressure reshapes the phonon spectral functions of low‐energy optical modes through peak shifts and linewidth broadening. This spectral‐function reshaping enhances their overlap with acoustic heat carriers, promotes acoustic–optical scattering, and shortens acoustic phonon lifetimes. These results identify low‐energy optical phonons as the key degrees of freedom through which chemical pressure controls thermal transport in SnTe.

## Graphical abstract

Chemical pressure reshapes low‐energy optical phonons in SnTe, altering their dispersion and scattering with heat‐carrying acoustic modes. Positive and negative pressure states modify phonon frequencies and lifetimes in distinct ways, revealing how mode‐selective lattice perturbations regulate acoustic–optical interactions and suppress lattice thermal conductivity beyond a simple volume effect in thermoelectric materials.

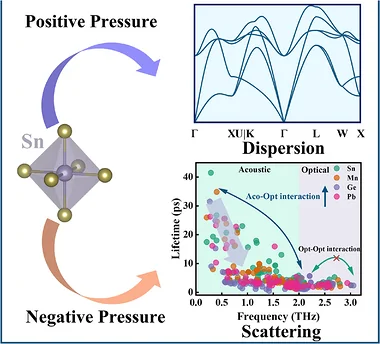

## 1 Introduction

Thermoelectric materials, enabling direct conversion between heat and electricity, have garnered immense potential for applications in power generation and solid‐state cooling [1, 2]. Their energy conversion efficiency is strongly constrained by thermal transport, particularly the lattice contribution to thermal conductivity, *κ*_l_, which is governed by phonon propagation [3, 4]. Suppressing *κ*_l_ through phonon engineering has thus become a significant strategy for improving thermoelectric performance [5, 6, 7]. Among these materials, SnTe is a PbTe‐analogous rock‐salt thermoelectric in which strong lattice anharmonicity and low‐energy optical modes play central roles in phonon transport [8, 9, 10]. Nevertheless, pristine SnTe still exhibits a relatively high lattice thermal conductivity [11, 12], suggesting that intrinsic anharmonicity alone does not fully suppress heat‐carrying phonons and that further control of phonon transport remains necessary in this system.

Pressure provides a powerful means of tuning phonon dynamics by modifying lattice volume, bond lengths, and interatomic force constants [13]. Physical pressure has therefore been widely used to investigate pressure‐dependent phonon renormalization, anharmonic scattering, and lattice thermal conductivity [14, 15, 16]. However, externally applied pressure is difficult to maintain in bulk thermoelectric materials under practical operating conditions, limiting its direct applicability in materials synthesis and device design. Chemical pressure, introduced through lattice‐site substitution, offers a practical alternative by internally perturbing the lattice while preserving ambient‐pressure stability [17, 18, 19]. This strategy has been widely used to regulate structural and functional properties in condensed‐matter materials and has recently attracted increasing attention in thermoelectric systems as a route for phonon engineering [20, 21, 22].

Despite its promise, how substitution‐induced chemical pressure microscopically modifies phonon transport remains insufficiently understood. In most cases, chemical pressure is quantified through lattice‐parameter changes or equivalent pressure values derived from an equation of state, which provides a useful macroscopic scale for comparing different compositions [17, 21, 23, 24]. However, such a scalar description does not directly reveal how substitution‐induced lattice perturbations modify local bonding environments, interatomic force constants, and the dynamics of individual phonon modes [16, 25]. Since phonon modes differ in their atomic displacement patterns and bonding characteristics, chemical pressure may not produce a uniform shift of the phonon spectrum. Instead, it can induce mode‐dependent phonon renormalization, reflected in changes in phonon frequencies, linewidths, and scattering channels [26]. This mode selectivity is particularly important in strongly anharmonic SnTe, where low‐lying optical modes couple to heat‐carrying acoustic phonons and thereby influence phonon scattering and *κ*_l_ [8, 27]. Clarifying these mode‐resolved responses is essential for understanding chemical‐pressure‐mediated phonon transport in SnTe‐based thermoelectrics.

In this work, Sn_0.95_M_0.05_Te (M = Sn, Mn, Ge, Sm, and Pb) is used as a model system to study chemical‐pressure‐mediated phonon transport in SnTe. The chemical pressure is calibrated from refined lattice parameters using the Birch–Murnaghan equation of state and is compared with physical‐pressure‐dependent Boltzmann transport calculations. Terahertz time‐domain spectroscopy, Raman spectroscopy, and Brillouin spectroscopy are used to resolve Γ‐point optical modes, L‐point optical modes, two‐phonon Raman features, and long‐wavelength acoustic phonons. These measurements are combined with first‐principles lattice‐dynamics calculations and spectral energy density analysis to analyze phonon frequency shifts, linewidth broadening, spectral‐weight redistribution, and lifetime changes under chemical pressure. These findings reveal how chemical pressure departs from a scalar lattice‐volume effect and enters phonon transport through the spectral‐function reshaping of low‐energy optical phonons.

## 2 Results and Discussion

To quantify chemical pressure, substitution‐induced lattice changes in SnTe are examined by X‐ray diffraction (XRD) and Rietveld refinement, as shown in Figure 1a,b. The XRD patterns of Sn_0.95_M_0.05_Te (M = Sn, Mn, Ge, Sm and Pb) samples are consistent with the face‐centered cubic rock‐salt structure of SnTe with space group *Fm*–3*m* (Powder Diffraction File Card No. 46–1210), with no detectable secondary phases. The refined lattice parameters exhibit systematic expansion or contraction depending on the Sn‐site substituent. Using pristine SnTe as the reference, the relative chemical pressure Δ*P*_chem_ is calibrated from these lattice parameters through the third‐order Birch–Murnaghan equation of state [28] and denotes the hydrostatic pressure required for pristine SnTe to reach the same average unit cell volume as the substituted sample. It is used only as a reference and does not represent the actual thermodynamic pressure or local mechanical stress in the substituted crystal. This calibration gives Δ*P*_chem_ values ranging from approximately −0.25 to +0.1 GPa, with M = Mn and Ge corresponding to positive chemical pressure and M = Sm and Pb corresponding to negative chemical pressure. Complementary scanning electron microscopy (SEM) and energy‐dispersive X‐ray spectroscopy (EDS) mapping on polished surfaces reveal dense microstructures and homogeneous elemental distributions for all compositions (Figure S1). X‐ray photoelectron spectroscopy (XPS) further confirms the presence of the substituted elements and their incorporation into the SnTe matrix within the detection limit of the measurement (Figure S1). These structural and compositional results define a chemically pressurized SnTe series with both positive and negative chemical‐pressure states.

**FIGURE 1:**
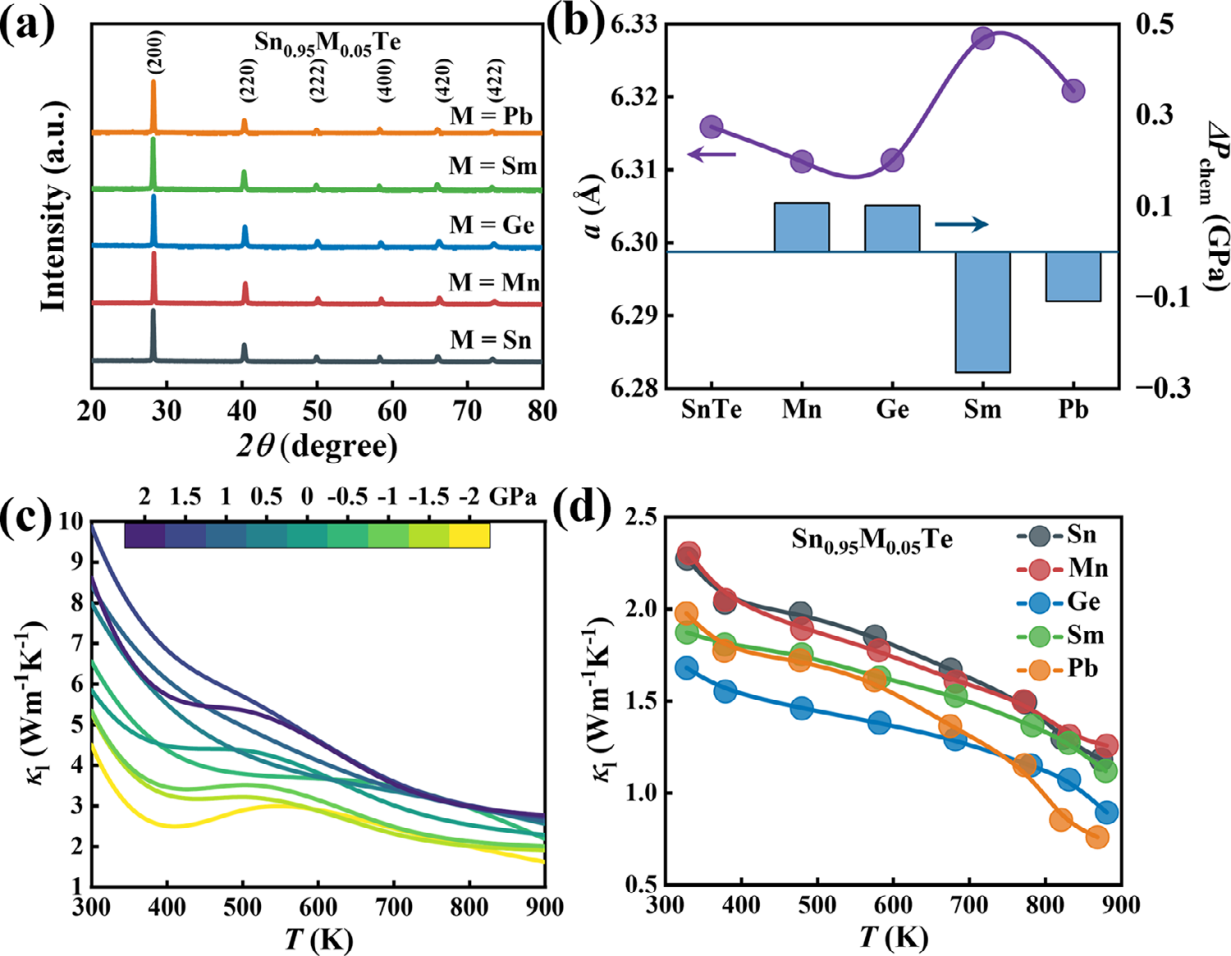
(a) XRD patterns for Sn_0.95_M_0.05_Te (M = Sn, Mn, Ge, Sm and Pb). (b) Lattice parameters obtained from Rietveld refinement and calibration of the chemical pressure difference based on the Birch‐Murnaghan third‐order equation of state (EOS), using the sample SnTe as the reference. (c) Lattice thermal conductivity of SnTe calculated using the Boltzmann transport equation (BTE) method as a function of physical pressure and temperature. (d) Lattice thermal conductivity for Sn_0.95_M_0.05_Te.

Homogeneous lattice expansion and compression are used to evaluate the pressure response of *κ*_l_ in SnTe. Figure 1c shows the lattice thermal conductivity of SnTe under physical pressure, obtained from first‐principles lattice dynamics combined with the Boltzmann transport equation over 300–900 K. The pressure dependence of *κ*_l_ is most pronounced at 300 K and gradually weakens at elevated temperatures. The stronger response near room temperature reflects the sensitivity of *κ*_l_ to pressure‐modified force constants and phonon dispersions. At elevated temperatures, enhanced anharmonic scattering reduces the contrast among different pressure states. Overall, *κ*_l_ increases under compression and decreases under lattice expansion. Positive pressure increases *κ*_l_, whereas lattice expansion, represented here by an equivalent negative pressure, lowers *κ*_l_. The latter is used only to describe the response of expanded SnTe. Against this physical‐pressure reference, thermal transport is measured for the Sn_0.95_M_0.05_Te samples. The electrical transport properties, including the Seebeck coefficient, electrical conductivity, and power factor, are summarized in Figure S2, together with the total thermal conductivity and the electronic contribution estimated from the Wiedemann–Franz law. The lattice contribution extracted from these data is shown in Figure 1d. Near room temperature, Sn_0.95_Ge_0.05_Te exhibits the strongest suppression of *κ*_l_, followed by the Sm‐ and Pb‐substituted samples, whereas Sn_0.95_Mn_0.05_Te shows a slightly higher *κ*_l_ than pristine SnTe. At higher temperatures, Sn_0.95_Pb_0.05_Te displays the most pronounced reduction and reaches the lowest *κ*_l_ among the series. The substituted samples exhibit distinct *κ*_l_ responses, setting up a comparison between physical and chemical pressure.

The further analysis begins with the physical‐pressure reference at 300 K, where *κ*_l_ shows the strongest pressure response. The BTE‐calculated values are plotted as scattered points in Figure 2a, while the solid line is obtained from the pressure‐dependent expression proposed by Tang [29],

(1)

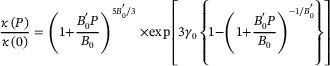

where *B*_0_, 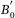, and *γ*_0_ are the bulk modulus, its first‐order pressure derivative, and the Grüneisen parameter at ambient pressure, respectively. The consistency between the BTE results and the Tang relation shows that the *κ*_l_ response to homogeneous physical pressure can be described by the elastic change of the SnTe lattice. This reference is then used to evaluate the thermal‐transport response under chemical pressure. The experimentally extracted *κ*_l_ values of Sn_0.95_M_0.05_Te are placed on the calibrated chemical‐pressure scale in Figure 2b. Except for the Mn‐substituted sample, the chemically pressurized samples deviate noticeably from the elastic response expected from volume change alone. The relative residuals from the Tang relation are −0.65%, −27.5%, −13.4%, and −11.3% for M = Mn, Ge, Sm, and Pb, respectively. Notably, M = Ge and Mn have nearly identical Δ*P*_chem_ values of 0.102 and 0.106 GPa, but their measured *κ*_l_ values are 1.679 and 2.304 W m^−1^ K^−1^, respectively. Thus, although Δ*P*_chem_ provides a useful macroscopic pressure scale, the thermal‐transport response under substitution‐induced pressure is not determined solely by lattice expansion or contraction.

**FIGURE 2:**
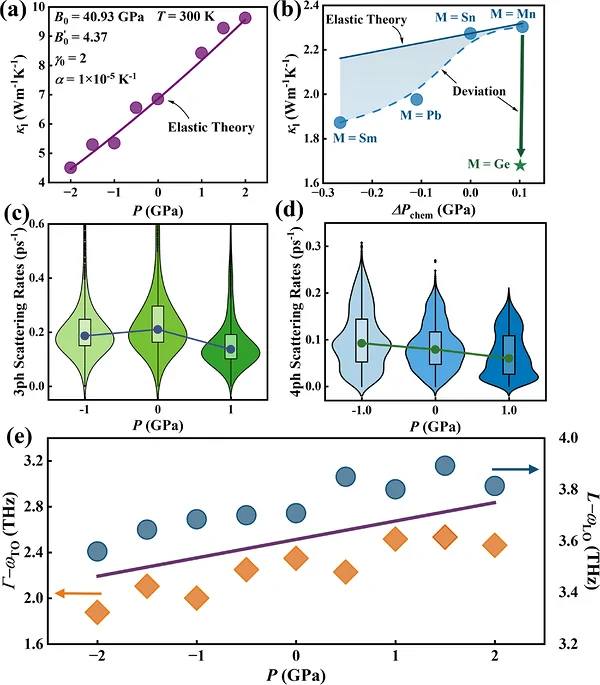
(a) The dependence of lattice thermal conductivity on physical pressure, as determined by the BTE method at 300 K (scatter) and calculated from the equation proposed by Tang (solid line), with the calculated parameters presented in the figure. (b) Dependence of lattice thermal conductivity on chemical pressure, where the blue solid lines are calculated from the equation based on elastic theory, and the green stars denote anomalous deviations in Sn_0.95_Ge_0.05_Te. The estimated standard uncertainties in *κ*_l_ are 0.12, 0.11, 0.13, 0.10, and 0.10 W m^−1^ K^−1^ for M = Sn, Mn, Ge, Sm, and Pb, respectively. Violin plot of (c) the three‐phonon scattering rate and (d) the four‐phonon scattering rate distribution of pristine SnTe under different physical pressures. (e) Dependence of the transverse optical (TO) phonon frequency at the Γ‐point and the longitudinal optical (LO) phonon frequency at the L‐point in the Brillouin zone on pressure.

The *κ*_l_ response to physical pressure can be traced to changes in phonon scattering and low‐energy phonon modes. Figure 2c,d show the pressure‐dependent distributions of three‐phonon and four‐phonon scattering rates calculated for pristine SnTe under homogeneous physical pressure. For three‐phonon processes, compression lowers the median scattering rate and suppresses the high‐scattering‐rate portion of the distribution, whereas lattice expansion produces a weaker change relative to ambient pressure. The four‐phonon scattering channel shows a more asymmetric pressure response, with lattice expansion broadening the distribution and increasing the population of large scattering rates, whereas compression shifts the distribution toward lower scattering rates. The observed enhancement of four‐phonon scattering under negative pressure aligns with previously reported findings [20]. These trends indicate that uniform lattice strain modifies the anharmonic scattering channels associated with *κ*_l_. The Γ‐point TO mode and the L‐point LO mode also exhibit systematic pressure‐dependent frequency shifts, as shown in Figure 2e. Both modes soften under lattice expansion and harden under compression, with an approximately monotonic dependence on pressure over the range. If chemical pressure were fully equivalent to this physical‐pressure reference, the measured *κ*_l_ would follow the same pressure‐controlled trend, together with corresponding changes in scattering rates and optical‐mode frequencies. Its deviation from this trend instead suggests that Δ*P*_chem_ does not capture the full phonon response to substitution‐induced lattice perturbation. For comparison with previous SnTe studies, near room‐temperature *κ*_l_ values for representative singly substituted SnTe systems are summarized in Table S1, together with their mass‐ and strain‐field fluctuation parameters. The unusually low *κ*_l_ of Ge‐substituted SnTe further suggests that its thermal‐transport response cannot be understood from conventional point‐ defect scattering alone. This motivates the investigation of Γ‐point optical modes, L‐point optical modes, and acoustic phonon lifetimes under chemical pressure.

Figure 3a maps the detection windows of THz‐TDS, Raman spectroscopy, and Brillouin spectroscopy onto the calculated phonon dispersion of SnTe, with representative displacement patterns shown alongside each probe. These techniques access complementary regions of momentum and frequency space. THz‐TDS probes the low‐frequency optical response near the Γ point [30], where the TO mode involves out‐of‐phase motion of Sn and Te atoms. Raman spectroscopy resolves optical modes away from the zone center, represented here by zone‐boundary vibrations [31], while Brillouin spectroscopy detects long‐wavelength acoustic phonons associated with collective in‐phase lattice motion [32]. For rock‐salt SnTe, the Γ‐point optical mode is infrared‐active but Raman‐inactive [33], making THz‐TDS the appropriate probe for the Γ‐point TO mode, with the optical‐constant extraction described in the Section 4. Figure 3b shows the THz‐TDS spectra of Sn_0.95_M_0.05_Te at 300 K. The measured and fitted Γ‐point TO peaks are shown in Figure S3, and the extracted TO‐mode frequencies and full widths at half maximum (FWHM) are summarized in Figure 3c. The TO frequencies do not follow the monotonic pressure evolution found under homogeneous physical pressure. Mn, Sm, and Pb substitution lead to moderate softening relative to pristine SnTe, whereas Ge substitution produces a much stronger frequency reduction. This pronounced softening may be associated with local ferroelectric‐like distortions around Ge, consistent with the ferroelectric instability known in GeTe‐based systems [34]. This interpretation is further supported by the 300 K AIMD analysis, in which the Ge‐centered environment exhibits substantially larger local off‐centering and dynamic displacements than the corresponding Sn‐ and Pb‐containing models (Table S2), indicating a stronger intrinsic tendency toward local polar distortion around Ge. The linewidths vary in the opposite direction to the mode frequencies, with softer TO modes generally exhibiting broader spectral features. This trend is most pronounced for Ge substitution, which shows the lowest TO frequency and the largest FWHM. For the damped phonon, the phonon spectral function is no longer a *δ*‐like peak at the harmonic frequency, but a broadened response whose peak position and linewidth describe the renormalized frequency and damping, respectively. The Ge‐substituted sample shows a strongly reshaped Γ‐point TO spectral function, combining pronounced softening with enhanced spectral broadening. The THz‐TDS results indicate that chemical pressure modifies the Γ‐point TO mode not only by shifting its frequency, but also by broadening its phonon spectral function.

**FIGURE 3:**
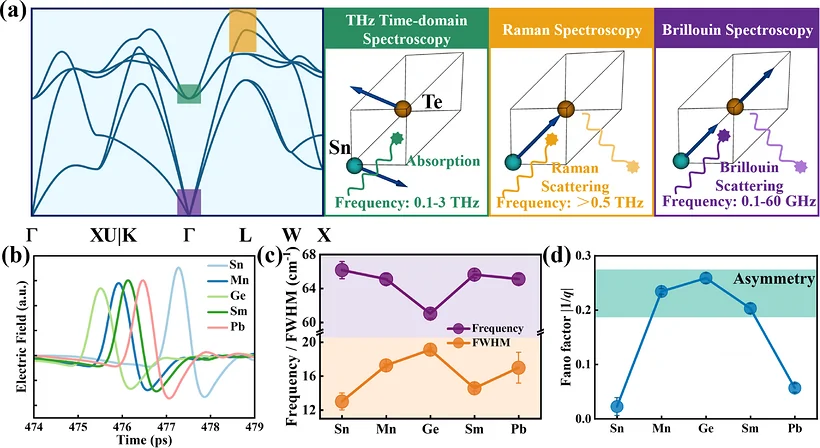
(a) Phonon dispersion relations of SnTe with superimposed detection windows for SnTe of three complementary techniques: THz time‐domain spectroscopy (THz‐TDS, green), Raman spectroscopy (yellow), and Brillouin spectroscopy (violet). The corresponding atomic displacement patterns are shown as insets. (b) The THz time‐domain spectra measured for Sn_0.95_M_0.05_Te at 300 K. The inset is a schematic diagram of the Γ‐point TO vibration mode for SnTe. (c) The frequency and full width at half maximum (FWHM) of the TO mode obtained by fitting THz time‐domain spectroscopy data. (d) The Fano factor fitted by the Breit‐Wigner‐Fano (BWF) model for Sn_0.95_M_0.05_Te, indicating the asymmetry of the peak shape. Error bars in (c) and (d) represent the uncertainties obtained from the spectral fitting.

Beyond the peak position and linewidth, the THz spectra contain information on the symmetry and coupling environment of the Γ‐point TO mode through their line shape. A Breit‐Wigner‐Fano profile, described in the Section 4, is used to quantify the asymmetric peak shape by the parameter 1/*q* [35]. Pristine SnTe shows a nearly symmetric phonon line shape, indicating negligible Fano‐type asymmetry. In contrast, Mn, Ge, and Sm substitution produce clear asymmetric profiles, whereas Pb substitution causes only a weak deviation from a symmetric response. This contrast indicates that chemical pressure changes the local symmetry and coupling environment of the TO mode through the specific lattice perturbation introduced by each Sn‐site substituent. The enhanced asymmetry in Mn‐, Ge‐, and Sm‐substituted samples indicates coupling between the discrete TO phonon and an overlapping continuum. Although the Fano line shape alone does not identify the microscopic origin of this continuum, its composition dependence is most consistent with a defect‐associated vibrational background [36, 37], since the asymmetry does not follow the electrical conductivity and pristine SnTe remains nearly symmetric despite its strong intrinsic anharmonicity. The weak asymmetry in the Pb‐substituted sample suggests that Pb incorporation perturbs the local symmetry less strongly than the other substitutions. Together with the frequency softening and linewidth broadening, these line‐shape changes show that chemical pressure selectively modifies the Γ‐point TO mode rather than producing a uniform pressure‐like shift of the phonon spectrum.

Raman spectroscopy is used to extend the mode‐resolved analysis from the Γ‐point TO mode to zone‐boundary optical phonons. Although the Γ‐point optical phonons of rock‐salt SnTe are Raman‐inactive by symmetry, defect‐activated Raman scattering can relax the momentum selection rule and make zone‐boundary optical phonons visible [38, 39]. Figure 4a shows the Raman spectra of Sn_0.95_M_0.05_Te, where P1 and P2 are assigned to the TO and LO phonon modes at the L point, respectively, and the broad P3 and P4 features are associated with a two‐phonon Raman process [33, 40]. The measured and fitted Raman spectra, including the decomposed P1, P2, P3, and P4 components, are shown in Figure S4. The fitted P1 and P2 frequencies in Figure 4b vary consistently with the sign of Δ*P*_chem_, hardening under positive chemical pressure and softening under negative chemical pressure. Mn and Ge substitution lead to slight hardening, while Sm and Pb substitution produce clear softening relative to pristine SnTe. This behavior is consistent with the sign of the calibrated chemical pressure and with the pressure‐induced optical‐mode shifts obtained under physical pressure. The linewidths in Figure 4c show a different behavior. The P1 mode broadens substantially for Mn, Ge, and Pb substitution, whereas the Sm‐substituted sample shows only a small change. The P2 mode follows a distinct trend, with the largest broadening observed for Sm substitution and a slightly narrower linewidth for Mn substitution than for pristine SnTe. The Raman results therefore show that Δ*P*_chem_ captures the main frequency shifts of the L‐point optical modes, whereas the linewidths are governed by mode‐specific phonon relaxation under substitution‐induced chemical pressure.

**FIGURE 4:**
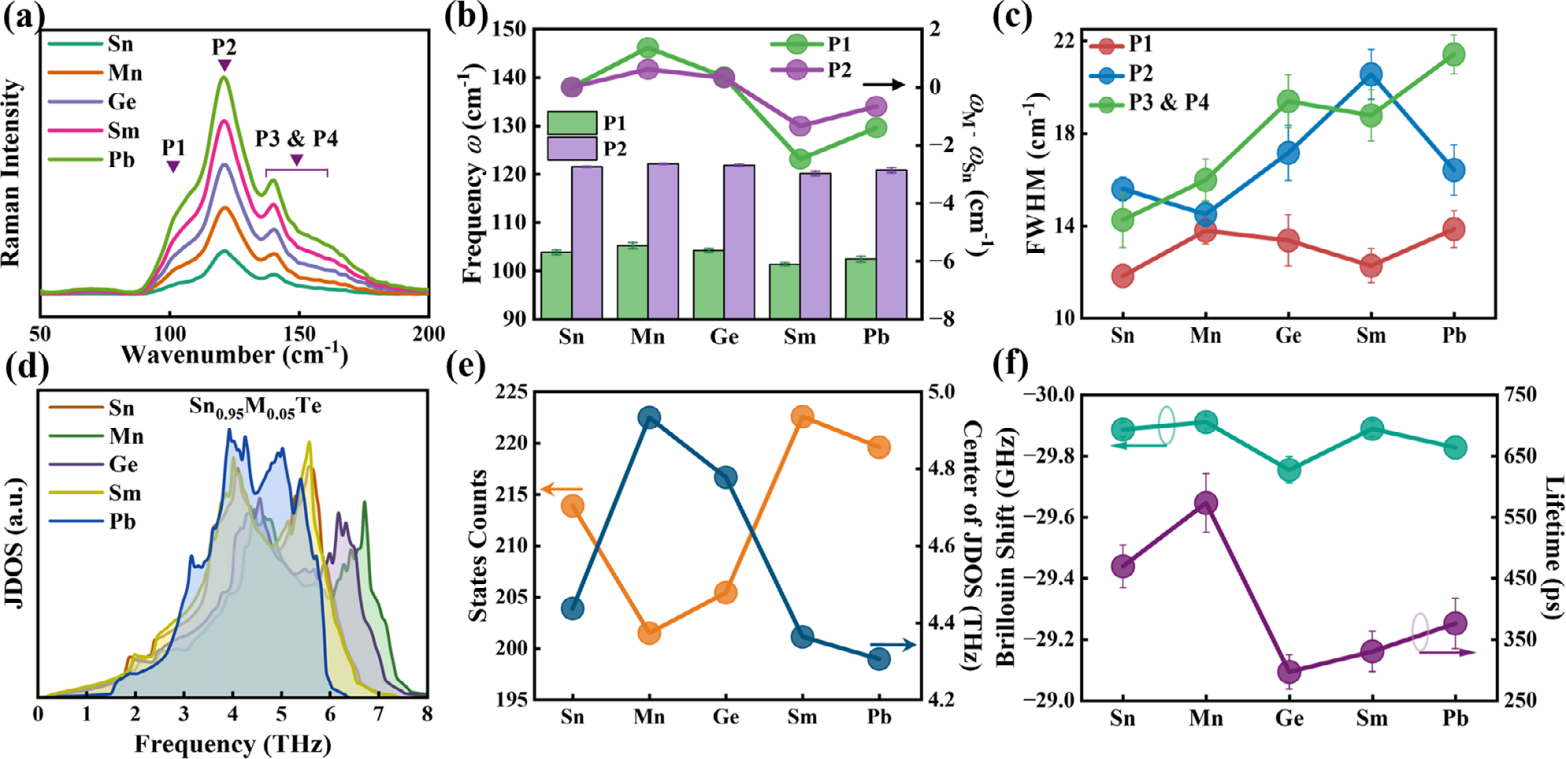
(a) Raman spectra of Sn_0.95_M_0.05_Te, with P1 and P2 marking the TO and LO phonon modes at the L points, respectively. The features labeled P3 and P4 correspond to a two‐phonon Raman scattering process. (b) Fitted frequencies of the P1 and P2 modes, and (c) the corresponding FWHM of P1 and P2, along with the weighted‐average FWHM values obtained from the two‐peak fitting of the P3&P4 feature. (d) Two‐phonon joint density of states (JDOS) for Sn_26_M_1_Te_27_ calculated using DFT. (e) Integrated counts of states derived from the JDOS shown in (d). (f) Brillouin frequency shifts and phonon lifetimes extracted from fitting the measured Brillouin spectra of Sn_0.95_M_0.05_Te, with the lifetimes determined from the corresponding FWHM. Error bars in (b, c) represent the uncertainties obtained from the Raman spectral fitting, while those in (f) represent the 16th–84th percentile intervals obtained from residual‐bootstrap analysis of the Brillouin spectra.

The broad P3&P4 feature is associated with two‐phonon Raman scattering and reflects phonon‐pair contributions beyond the first‐order L‐point modes [40]. It is fitted with two peaks to describe the profile, rather than to assign two independent first‐order phonon modes. The weighted‐average FWHM of this feature increases for all substituted samples, with the weakest change for Mn and the strongest broadening for Pb, as shown in Figure 4c. Here, the weighted‐average FWHM is used only as a descriptive measure of the overall width of the P3&P4 spectral envelope. To evaluate the phase space underlying this two‐phonon response, the two‐phonon joint density of states is calculated using DFT for Sn_26_M_1_Te_27_, as shown in Figure 4d. The JDOS counts phonon‐pair states whose combined frequency contributes to a given Raman shift while approximately satisfying the momentum‐compensation condition *q*_1_+*q*_2_ ≈ 0. The pristine configuration shows two main JDOS features near 4 and 5.5 THz. Mn and Ge substitution shift the distribution toward higher frequencies, while Pb substitution gives a more confined distribution. The integrated counts in Figure 4e further show that positive chemical pressure reduces the number of momentum‐compensated phonon pairs, whereas negative chemical pressure increases it. In the Fermi's golden‐rule description, phonon scattering rates depend on anharmonic matrix elements, phonon occupation factors, and a kinematic phase‐space factor set by energy and momentum conservation [41, 42]. The two‐phonon JDOS captures this kinematic factor for the Raman‐active two‐phonon response by counting phonon‐pair states with nearly compensated momenta [43, 44]. A larger integrated JDOS therefore indicates a denser set of momentum‐compensated phonon‐pair states sampled by the two‐phonon Raman response, reflecting a redistribution of the available multiphonon phase space.

To relate these optical and multiphonon signatures to heat transport, the long‐wavelength acoustic phonons are probed by Brillouin spectroscopy. Brillouin spectroscopy resolves acoustic phonon peaks near 30 GHz, assigned to longitudinal acoustic phonons based on their consistency with the calculated sound velocity. The measured and fitted LA phonon peaks are shown in Figure S5, and the extracted Brillouin frequency shifts and phonon lifetimes are summarized in Figure 4f. The acoustic frequencies change only weakly across the series, indicating that low‐level substitution has a limited effect on the long‐wavelength acoustic velocity. The lifetimes, however, vary much more strongly. Mn substitution gives a longer lifetime of approximately 550 ps, whereas Ge substitution produces the shortest lifetime of about 300 ps. Sm‐ and Pb‐substituted samples also show reduced lifetimes of approximately 350 ps. The weak variation in acoustic frequency, together with the strong variation in lifetime, indicates that chemical pressure affects heat‐carrying acoustic phonons mainly through relaxation processes rather than through changes in sound velocity. Together, the THz, Raman, and Brillouin spectra results show that the influence of chemical pressure on *κ*_l_ is carried by several mode‐resolved quantities, including Γ‐ and L‐point optical linewidths, multiphonon phase space, and acoustic phonon lifetimes, rather than by a single frequency or volume parameter.

To assess the spectroscopic descriptors as a whole, the measured phonon features are compared with *κ*_l_ in an exploratory correlation network, as shown in Figure S6. Because the composition series is limited in size, the network is used only as a descriptive map rather than a statistical model. A physically consistent tendency is nevertheless observed. Frequency‐related descriptors generally correlate positively with *κ*_l_, while linewidth‐related descriptors mostly correlate negatively. The clearest trends involve the linewidth of the longitudinal acoustic phonon and the L‐point LO mode. The former is directly related to the lifetime of heat‐carrying acoustic phonons, whereas the latter reflects broadening of a low‐lying optical branch that may participate in acoustic phonon relaxation by acoustic‐optical coupling. These correlations support the spectroscopic picture that *κ*_l_ is more closely associated with phonon relaxation than with a single pressure or frequency parameter. They also motivate further theoretical analysis of how chemical pressure modifies the microscopic phonon states underlying these spectral signatures.

To further elucidate the influence of chemical pressure on phonon dynamics, lattice dynamics calculations are performed to complement and corroborate the spectroscopic findings. Phonon dispersions are computed using the Sn_26_M_1_Te_27_ supercell configuration. This single‐substitution configuration is used as a controlled dilute‐substitution model to isolate the mode‐specific response introduced by each substituent. Given that the application of a supercell leads to Brillouin zone folding and a concomitant increase in the number of phonon branches, phonon unfolding techniques and temperature renormalization methods are employed to recover the effective phonon spectra and corresponding spectral weights. The resulting phonon dispersions and mode‐dependent spectral intensities for Sn_26_M_1_Te_27_ are presented in Figure 5. First, it can be observed that, with the exception of Sn_26_Pb_1_Te_27_, all other substituted configurations exhibit distinct spectral features arising from symmetry breaking, manifesting as discrete lines in the unfolded phonon spectra. These additional spectral features arise from reduced translational symmetry and enhanced mode localization in the substituted configurations. In contrast, the Pb‐substituted configuration exhibits much weaker additional spectral features than the Mn‐, Ge‐, and Sm‐substituted cases. This observation is consistent with the analysis of the Fano asymmetry parameter for the Γ‐point TO mode. Notably, in the Sn_26_Ge_1_Te_27_ configuration, a prominent soft mode emerges below the Γ‐point TO phonon, which can be associated with the local ferroelectric distortions induced by Ge substitution. Furthermore, the phonon frequencies exhibit notable hardening for the Γ‐point TO mode in the Sn_26_Mn_1_Te_27_ configuration. In contrast, configurations subjected to negative chemical pressure display substantial softening across all phonon modes, with the effect being more pronounced in Sn_26_Pb_1_Te_27_. These calculated phonon frequencies and their responses to chemical pressure are in good agreement with the spectroscopic results presented above. The calculated spectra identify branch‐selective spectral‐weight redistribution as the microscopic origin of the nonuniform phonon responses observed under chemical pressure.

**FIGURE 5:**
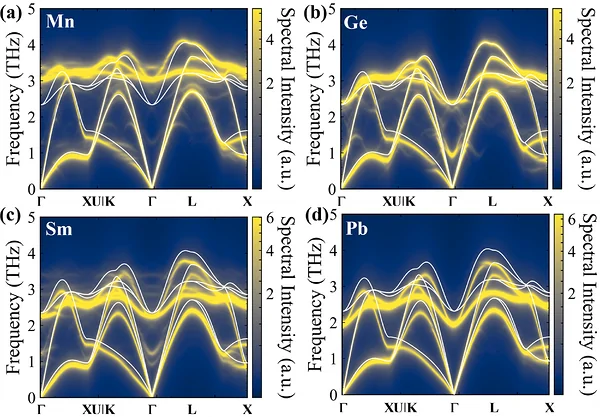
Phonon band structure of configurations for (a) M = Mn, (b) M = Ge, (c) M = Sm, and (d) M = Pb calculated using the mode decomposition method. The phonon dispersion plotted with a solid white line is calculated using the primitive cell of SnTe and is shown for comparison.

The mode‐decomposition analysis identifies branch‐selective redistribution of phonon spectral weight, but the associated phonon relaxation still needs to be quantified. Pristine SnTe is included as the reference, while Mn‐, Pb‐, and Ge‐substituted configurations are selected to represent positive chemical pressure, negative chemical pressure, and the anomalous response, respectively. Spectral energy density analysis is therefore carried out using molecular‐dynamics trajectories generated from the validated neuroevolution potential, whose energy, force, and virial accuracy are summarized in Figure S8. The resulting SED maps are shown in Figure S10, and representative Lorentzian fits at the Γ and L points are given in Figures S11 and S12. Each fitted SED peak can be viewed as a mode‐resolved phonon spectral function, where the peak position gives the renormalized frequency, and the linewidth reflects damping associated with the finite phonon lifetime. The phonon branches also remain relatively well separated, with individually resolvable SED peaks, consistent with a predominantly quasiparticle description. This differs from strongly anharmonic or structurally complex systems with densely distributed vibrational states, where nonpropagating or coherence contributions can become appreciable [45, 46]. The fitted FWHM values are summarized in Figure 6a for the Γ‐point mode and three resolved L‐point phonon peaks, denoted L1, L2 and L3. The linewidths do not evolve uniformly across these modes. The Γ‐point linewidth increases from pristine SnTe to the Ge‐substituted configuration and decreases slightly for Pb, whereas the three L‐point peaks show separate trends. L2 and L3 have broaden progressively across the substituted configurations, while L1 narrows for Mn and Ge but broadens again for Pb. This redistribution of linewidth among Γ‐ and L‐point modes shows that chemical pressure changes phonon relaxation selectively across the low‐energy optical branches. The lifetimes extracted from all fitted *q* points are summarized as a function of frequency in Figure 6b. Long lifetimes are mainly retained in the low‐frequency acoustic region, consistent with acoustic phonons acting as the dominant heat carriers. The lifetime distribution, however, changes substantially among the substituted configurations. In SnTe, low‐lying optical branches lie close to the acoustic spectrum, making acoustic–optical scattering an important relaxation pathway for heat‐carrying acoustic phonons [8, 9, 47]. Chemical pressure changes this pathway by reshaping the phonon spectral functions of low‐energy optical modes. Frequency shifts change the energy separation between low‐energy optical modes and acoustic heat carriers, while linewidth broadening spreads the optical modes over a wider frequency range [48]. Together, these changes enhance acoustic–optical spectral overlap, promote scattering between optical modes and acoustic heat carriers, and shorten acoustic phonon lifetimes. The mode‐decomposition and SED results show that these two effects occur unevenly among the low‐energy optical branches, with negative chemical pressure mainly acting through optical‐mode softening and Ge substitution through pronounced TO spectral broadening. Therefore, chemical pressure controls *κ*_l_ through the spectral functions of low‐energy optical modes that govern acoustic phonon relaxation, rather than by a rigid pressure‐like shift of the phonon dispersion.

**FIGURE 6:**
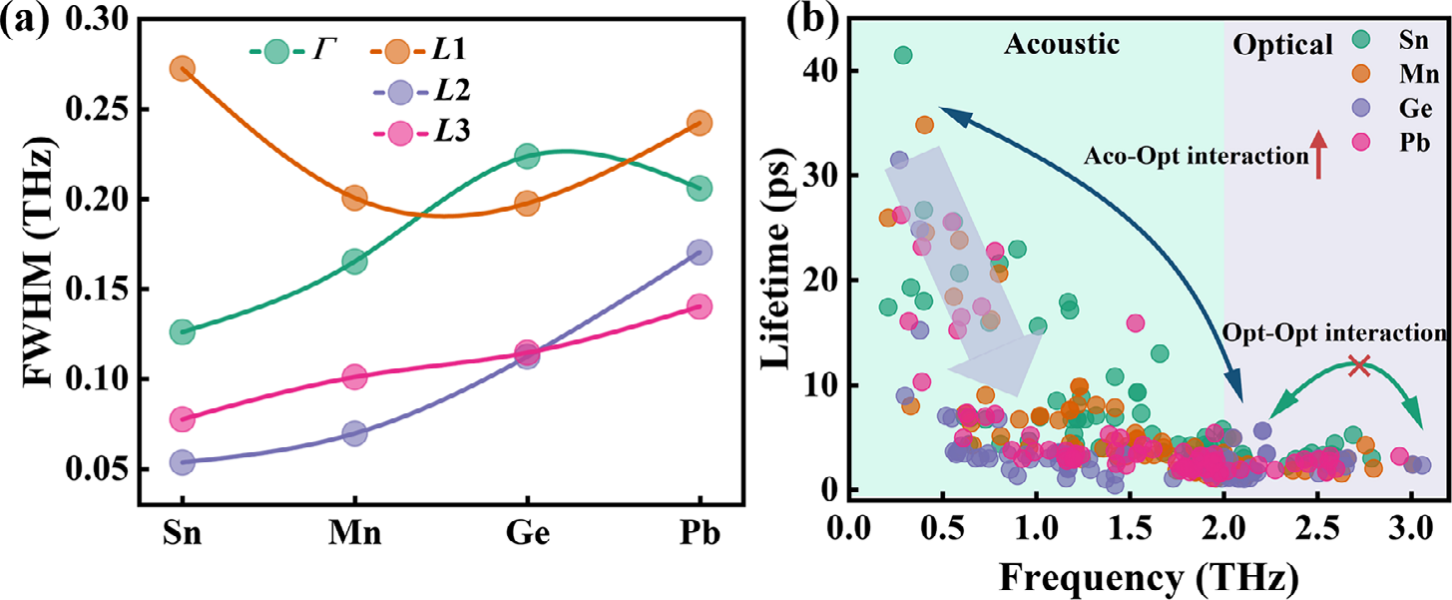
(a) Full width at half maximum (FWHM) of spectral energy density (SED) fitted at the Γ point and L points (three peaks, denoted as L1, L2 and L3) in the Brillouin zone for M = Sn, Mn, Ge, and Pb. (b) Phonon lifetimes as a function of frequency extracted from SED fitting of all q‐points.

## 3 Conclusion

This work reveals how chemical pressure regulates phonon transport in SnTe beyond the scalar lattice‐volume description. Physical pressure provides a homogeneous strain reference, where κl follows an elasticity‐based pressure dependence with regular changes in anharmonic scattering and optical‐mode frequencies. In contrast, Sn‐site substitution produces chemical pressure that only partly follows this macroscopic trend. The measured *κ*_l_ values deviate from a single pressure‐controlled relation, indicating that substitution‐induced lattice perturbations introduce phonon responses not captured by Δ*P*_chem_ alone. Mode‐resolved spectroscopy and lattice‐dynamics calculations show that chemical pressure modifies the Γ‐point TO mode, L‐point optical modes, the JDOS associated with two‐phonon Raman processes, and acoustic phonon lifetimes with different sensitivities. Frequency shifts retain a partial pressure‐like character, whereas linewidth broadening, Fano asymmetry, JDOS redistribution, and acoustic lifetime changes are more sensitive to local lattice perturbations. Lattice‐dynamics and SED analysis further show that chemical pressure reshapes the phonon spectral functions of low‐energy optical modes, with peak shifts and linewidth broadening jointly controlling their overlap with acoustic heat carriers. Optical‐mode softening and spectral broadening provide two spectral routes to enhanced acoustic phonon relaxation and reduced *κ*_l_. These findings provide a useful framework for engineering lattice thermal transport in materials with low‐lying optical branches near the acoustic spectrum, including cubic IV–VI thermoelectrics.

## 4 Methods

### 4.1 Experimental Details

#### 4.1.1 Materials Synthesis

High‐purity single elements Sn (99.99%, Alfa Aesar), Te (99.99%, Alfa Aesar), Mn (99.95%, Aladdin), Ge (99.9%, Aladdin), Sm (99.9%, Aladdin), Pb (99.99%, Alfa Aesar) were weighed according to the stoichiometric ratio, and the mixtures were sealed in quartz tubes after weighing. The specimens were subjected to the following thermal schedule: heating from 323 to 923 K for 720 min, isothermal holding at 923 K for 120 min, heating to 1473 K for 360 min, maintaining at 1473 K for 360 min, and subsequent quenching to ambient temperature. The resulting ingots were hand‐milled into fine powder, and the powders were densified by Rapid Hot Pressing at 823 K and 50 MPa for 10 min. This process produced highly dense discoidal pellets with a diameter of 12.7 mm and a thickness of 2 mm.

#### 4.1.2 Material Characterization

Powder X‐ray diffraction (XRD) patterns were obtained using a Rigaku Smart Lab 3KW diffractometer with Cu K𝛼 radiation (𝜆 = 1.5418 Å). Rietveld refinements were conducted using GSASII software. Scanning electron microscopy (SEM) and energy‐dispersive spectroscopy (EDS) were performed with a JSM‐7610F scanning electron microscope. X‐ray photoelectron spectroscopy (XPS) was conducted on a Thermo Scientific ES CALAB 250 Xi spectrometer with a monochromatic Al K𝛼 X‐ray source (1486.6 eV) and an operating power of 300 W. Electrical conductivity and Seebeck coefficient measurements, ranging from 323 to 873 K, were conducted using a Linseis LSR‐3 instrument. Thermal diffusivity coefficients (*λ*) were measured using a TA Instruments DLF 1200 via the laser flash method. Density (*d*) was determined by the Archimedes method, while the heat capacity (*C_p_*) was calculated using the Debye model. The total thermal conductivity (*κ*) was calculated using the formula *κ* = *λ C_p_ d*. The standard measurement uncertainty in *κ*_l_ was estimated by Type B propagation of the instrumental repeatability in thermal diffusivity, electrical conductivity, and Seebeck coefficient through κ_*l*_  =  *DC*_p_ρ  − *L*σ*T*, with the uncertainty in *L* propagated from the Seebeck coefficient. The THz time‐domain spectroscopy was conducted on TERASCAN 1550, a Terahertz Spectroscopy System with High Spectral Resolution. Raman spectra were obtained using a inVia Qontor system with a 532 nm laser. Brillouin spectroscopy was performed using a custom‐built Fabry–Pérot interferometer, also utilizing a 532 nm laser. The THz, Raman, and Brillouin spectra were fitted using Breit–Wigner–Fano, Voigt, and Lorentzian profiles, respectively. Uncertainties in the THz and Raman parameters were obtained from the spectral fits, while those for the Brillouin frequency shifts and phonon lifetimes were determined by residual‐bootstrap refitting using the 16th–84th percentile ranges. To examine measurement reproducibility, spectral measurements were repeated on independently synthesized SnTe and Sn_0.95_Ge_0.05_Te samples, showing consistent spectral profiles and closely matched fitted phonon parameters (Figure S7).

#### 4.1.3 THz‐TDS Optical Constants Analysis

THz time‐domain spectroscopy directly measures the transmitted electric field in both amplitude and phase. The complex optical response of the sample can therefore be obtained without relying on Kramers‐Kronig transformation [49]. The refractive index *n*(*ω*) and absorption coefficient *α*(*ω*) are calculated from the reference and sample signals as:

(2) $$n\;\left(\omega \right)=1+\frac{c}{\omega L}\left[\phi_{\mathrm{Ref}}\left(\omega \right)-\phi_{\mathrm{Samp}}\left(\omega \right)\right]$$

(3) $$\alpha \;\left(\omega \right)=-\frac{2}{L}\ln\left(\frac{{\left[n\left(\omega \right)+1\right]}^{2}}{4n\left(\omega \right)}\cdot \left|\frac{E_{\mathrm{Samp}}(\omega )}{E_{\mathrm{Ref}}(\omega )}\right|\right)$$

where *ϕ*_Samp_ and *ϕ*_Ref_ denote the phases of the sample and reference signals, respectively; *E*_Samp_ and *E*_Ref_ are the corresponding amplitudes, *ω* is the angular frequency, *c* is the speed of light in vacuum, and *L* is the sample thickness. The asymmetric absorption peak of the Γ‐point TO mode is fitted using the Breit‐Wigner‐Fano line shape [36],

(4) $$I\left(\omega \right)=I_{0}\frac{{\left[q+2\left(\omega -\omega_{0}\right)/\Gamma \right]}^{2}}{1+{\left[2\left(\omega -\omega_{0}\right)/\Gamma \right]}^{2}}$$

where *ω*_0_ is the peak center frequency, *Γ* represents the linewidth, *I*_0_ denotes the peak intensity, and the Fano parameter *q* quantifies the degree of asymmetry. As *q* approaches unity, the asymmetry becomes more pronounced, whereas in the limit *q*→∞, the line shape converges to a symmetric Lorentzian profile.

### 4.2 Computational Details

First‐principles calculations were performed based on density functional theory (DFT) as implemented in the Vienna Ab initio Simulation Package (VASP) [50, 51, 52]. The exchange‐correlation functional was treated within the generalized gradient approximation (GGA) using the PBEsol parametrization [53]. The projector‐augmented wave (PAW) method [54, 55] was employed with valence electronic states expanded in a plane‐wave basis set with a kinetic energy cutoff of 500 eV. Structural relaxations were performed until the Hellmann‐Feynman forces on each atom were less than 10^−4^ eV/Å, and the total energy convergence criterion was set to 10^−8^ eV. A Γ‐centered Monkhorst‐Pack k‐point mesh with a grid density of 0.15 Å^−1^ was used for Brillouin zone sampling.

To investigate the effects of physical pressure on the lattice thermal conductivity, a series of configurations were generated. The equilibrium structural parameters at various pressures were determined by fitting the calculated energy‐volume data to the third‐order Birch‐Murnaghan equation of state (EOS). The temperature‐dependent effective interatomic force constants (IFCs) were extracted using the temperature‐dependent effective potential (TDEP) method [56, 57] combined with ab initio molecular dynamics (AIMD) simulations. AIMD calculations were performed in the canonical (NVT) ensemble using the Nosé‐Hoover thermostat, covering a temperature range of 300–900 K with intervals of 100 K. For each temperature point, a 4 × 4 × 4 supercell was simulated for 10 ps with a time step of 1 fs. The second‐, third‐, and fourth‐order effective force constants were determined by a least‐squares fitting procedure using the TDEP method. The lattice thermal conductivity and related phonon transport properties were calculated by solving the phonon Boltzmann transport equation (BTE) using the FourPhonon package, which is developed based on the ShengBTE framework [41, 58]. A dense 30 × 30 × 30 q‐point mesh was used for the Brillouin zone integration to ensure convergence of the thermal conductivity values. For the calculation of the four‐phonon scattering rate, a sampling technique [59] was employed with a sample size of 500 000.

Through the substitution of a single Sn atom within the 3 × 3 × 3 supercell, a series of chemical pressure‐induced configurations were generated, followed by structural relaxation. For these chemical pressure configurations, the interatomic force constants were calculated using the finite displacement method as implemented in Phonopy [42, 60]. To capture the temperature‐dependent anharmonic renormalization of phonon frequencies, AIMD simulations were performed at 300 K as described above. The trajectories were subsequently analyzed using the Dynaphopy package [61], which implements the normal mode decomposition method. The atomic velocities from AIMD were projected onto the harmonic normal mode coordinates obtained from Phonopy calculations, and the renormalized second‐order force constants were extracted. The weighted joint density of states (JDOS) was calculated using the phono3py package [42], with the method as follows:

(5) $$N\;\left(q,\omega \right)=\frac{1}{N}\;\sum_{\lambda^{\prime }\lambda^{\prime \prime }}\Delta \left(-q+q^{\prime }+q^{\prime \prime }\right)\left(n_{\lambda^{\prime }}+n_{\lambda^{\prime \prime }}+1\right)\delta \left(\omega -\omega_{\lambda^{\prime }}-\omega_{\lambda^{\prime \prime }}\right)$$

Due to the symmetry reduction induced by substitution, the phonon modes fold from the primitive Brillouin zone into the reduced supercell zone. To recover the effective phonon dispersion in the primitive Brillouin zone (BZ) and visualize the chemical pressure‐induced spectral broadening, the phonon spectral function *A*_s_(**K**,*ω*) was calculated using the Upho package [62]. The unfolding procedure projects the supercell vibrational modes onto the primitive cell reciprocal lattice via the spectral weight method:

(6) $$A_{s}\;\left(K,\omega \right)=\sum_{k=1}^{N}A\left(k_{k},\omega \right)=\sum_{k=1}^{N}\sum_{J}{\left|{\left[{\hat{P}}^{k_{k}}\tilde{v}\left(K,J\right)\right]}_{l}\right|}^{2}\delta \left[\omega -\omega \left(K,J\right)\right]$$

The spectral function *A*_s_(**K**,*ω*) defined in the BZ for the supercell model is remapped in the BZ for the underlying crystal structure with the weights obtained from ${\left[{\hat{P}}^{k_{k}}\tilde{v}\left(K,J\right)\right]}_{l}$.

The Graphics Processing Unit Molecular Dynamics (GPUMD) software package employs molecular dynamics with the Neural Evolutionary Potential (NEP) [63, 64]. The training dataset for this machine‐learning interatomic potential consists of energies and atomic forces derived from DFT calculations. In constructing the NEP model, the radial and angular cutoff radii are set to 8 and 4 Å, respectively. The radial and angular descriptor components contain 5 and 25 elements, respectively, both with a base length of 8, and the hidden layer is configured with 60 neurons. A total of 17851 parameters are optimized during training, with the weight factors for energy loss, force loss, and virial loss set to 1.0, 1.0, and 0.1, respectively. The model parameters converge after 42 300 iterations. Ultimately, the root‐mean‐square errors (RMSEs) on the training set are 7.95 meV/atom for energy, 147.92 meV/Å for force, and 0.2802 GPa for virial, which are shown in Figure S8. The transferability of the NEP model was further assessed using an independent substituted test set and by direct comparison of the DFT and NEP unfolded phonon spectra; the corresponding results are summarized in Figure S9 and Table S3, respectively. A 20 × 20 × 20 supercell containing 16 000 atoms was used as the initial model. Substitutional atoms were introduced at a concentration of 5% with a random distribution. The preparation protocol consisted of three successive equilibration stages, followed by a production run. First, the system was relaxed in the isothermal–isobaric (NPT) ensemble for 1 ns at 300 K and ambient pressure using a stochastic cell‐rescaling barostat. Second, the pressure control was removed and the system was further equilibrated in the canonical (NVT) ensemble for another 1 ns at 300 K, employing the Nosé–Hoover chain thermostat. Finally, the thermostat was turned off, and a production run of 500 ps was performed in the microcanonical (NVE) ensemble. During production, atomic coordinates and velocities were dumped every 50 fs. The velocity trajectories were analyzed with the pySED package using the spectral energy density (SED) method to obtain phonon dispersion relations and lifetimes [65, 66].

## Author Contributions

**Zhihao Li**: conceptualization, methodology, investigation, validation, formal analysis, writing – original draft, writing – review and editing. **Jianhong Hu**: investigation, formal analysis. **Panpan Peng**: investigation, validation. **Peng Cao**: investigation, validation. **Hui Zhang**: investigation, formal analysis. **Yujie Zong**: formal analysis, visualization. **Yanhui Wu**: visualization, investigation. **Hongchao Wang**: conceptualization, methodology, project administration, resources, supervision, writing – review and editing, writing – original draft.

## Funding

This work was funded by the National Natural Science Foundation of China under Grant Nos. 52472221 and W2521091, the SCO Science and Technology Partnership Program under Grant No, 2026E01005 and the Core Facility Sharing Platform of Shandong University.

## Conflicts of Interest

The authors declare no conflicts of interest.

## Supporting information




**Supporting file**: advs78136‐sup‐0001‐SuppMat.pdf.

## Data Availability

The data that support the findings of this study are available from the corresponding author upon reasonable request.
