# Supplementary material for: Mode‐Resolved Phonon Dynamics Under Chemical Pressure in SnTe Thermoelectrics
Source: Adv Sci (Weinh). 2026 Oct 6:e78136. Online ahead of print. doi: 10.1002/advs.78136 (PMC13643105; doi:10.1002/advs.78136)
Supplement: Supplementary file 1 [file ADVS-9999-e78136-s001.pdf]

Supporting Information

Mode-Resolved Phonon Dynamics under Chemical Pressure in SnTe Thermoelectrics

*Zhihao Li, Jianhong Hu, Panpan Peng, Peng Cao, Hui Zhang, Yujie Zong, Yanhui Wu, Hongchao Wang**

School of Physics, Shandong University, Jinan, 250100, PR China

E-mail: wanghc@sdu.edu.cn

Table S1. Comparison of the lattice thermal conductivity and calculated point-defect scattering parameters for singly substituted SnTe near room temperature.

**Composition**	***T* (K)**	**κ_l (W m^{-1} K^{-1})**	**Γ_M+Γ_S (10^{-2})**	**Reference**
$Sn_{0.95}Co_{0.05}Te$	323	2.05	6.145	[1]
$Sn_{0.95}Ni_{0.05}Te$	323	2.73	6.192	[1]
$Sn_{0.95}Zn_{0.05}Te$	323	3.05	4.964	[1]
$Sn_{0.95}Ge_{0.05}Te$	323	1.76	5.056	[1]
$Sn_{0.97}Mn_{0.03}Te$	300	2.95	4.026	[2]
$Sn_{0.95}Mn_{0.05}Te$	300	2.25	6.639	[2]
$Sn_{0.98}Mg_{0.05}Te$	300	2.10	14.187	[3]
**$Sn_{0.95}Mn_{0.05}Te$**	**323**	**2.30**	**6.639**	**This work**
**$Sn_{0.95}Ge_{0.05}Te$**	**323**	**1.68**	**5.056**	**This work**
**$Sn_{0.95}Sm_{0.05}Te$**	**323**	**1.86**	**8.174**	**This work**

[1] M. Aminzare, Y.-C. Tseng, A. Ramakrishnan, K.-H. Chen, and Y. Mozharivskyj, Effect of single metal doping on the thermoelectric properties of SnTe, Sustainable Energy & Fuels 3 (2019) 251–263.

[2] X. Xu, J. Cui, L. Fu, Y. Huang, Y. Yu, Y. Zhou, D. Wu, and J. He, Enhanced Thermoelectric Performance Achieved in SnTe via the Synergy of Valence Band Regulation and Fermi Level Modulation, ACS Applied Materials & Interfaces 13 (2021) 50037–50045.

[3] A. Banik, U. S. Shenoy, S. Anand, U. V. Waghmare, and K. Biswas, Mg Alloying in SnTe Facilitates Valence Band Convergence and Optimizes Thermoelectric Properties, Chemistry of Materials 27 (2015) 581–587.

The point-defect scattering strength is estimated using the mass- and strain-field fluctuation parameters. The mass fluctuation parameter Γ_M describes the variance in atomic mass on the substituted Sn site, while the strain-field parameter Γ_S accounts for the local lattice distortion arising from the size mismatch between the host and substituent atoms. The total fluctuation parameter was taken as $\Gamma_M + \Gamma_S$.

Table S2. Local cation off-centering and dynamic relative displacements derived from the 300 K ab initio molecular-dynamics trajectories.

MD model	RMS time-averaged offset (Å)	RMS dynamic displacement (Å)
SnTe	0.013	0.254
$Sn_{0.75}Ge_{0.25}Te$	0.045	0.333
$Sn_{0.75}Pb_{0.25}Te$	0.006	0.210

Local cation displacements are evaluated from the 300 K AIMD trajectories. The local offset is defined as the displacement of each cation from the center of its nearest-neighbor Te coordination cage, and the RMS local offset is obtained by averaging this quantity over the trajectory. The RMS dynamic displacement is calculated from the fluctuation of each atomic position around its time-averaged position.

Table S3. Agreement between the DFT and NEP unfolded phonon spectra quantified by the q-resolved first-order Wasserstein distance, W_1, which measures the spectral displacement between the two normalized phonon spectral distributions.

**System**	**Mean W_1 (THz)**	**Median W_1 (THz)**	**95th-percentile W_1 (THz)**
Sn	0.1183	0.1147	0.1634
Mn	0.1178	0.0993	0.1689
Ge	0.1332	0.1241	0.1713
Pb	0.1277	0.1170	0.1536

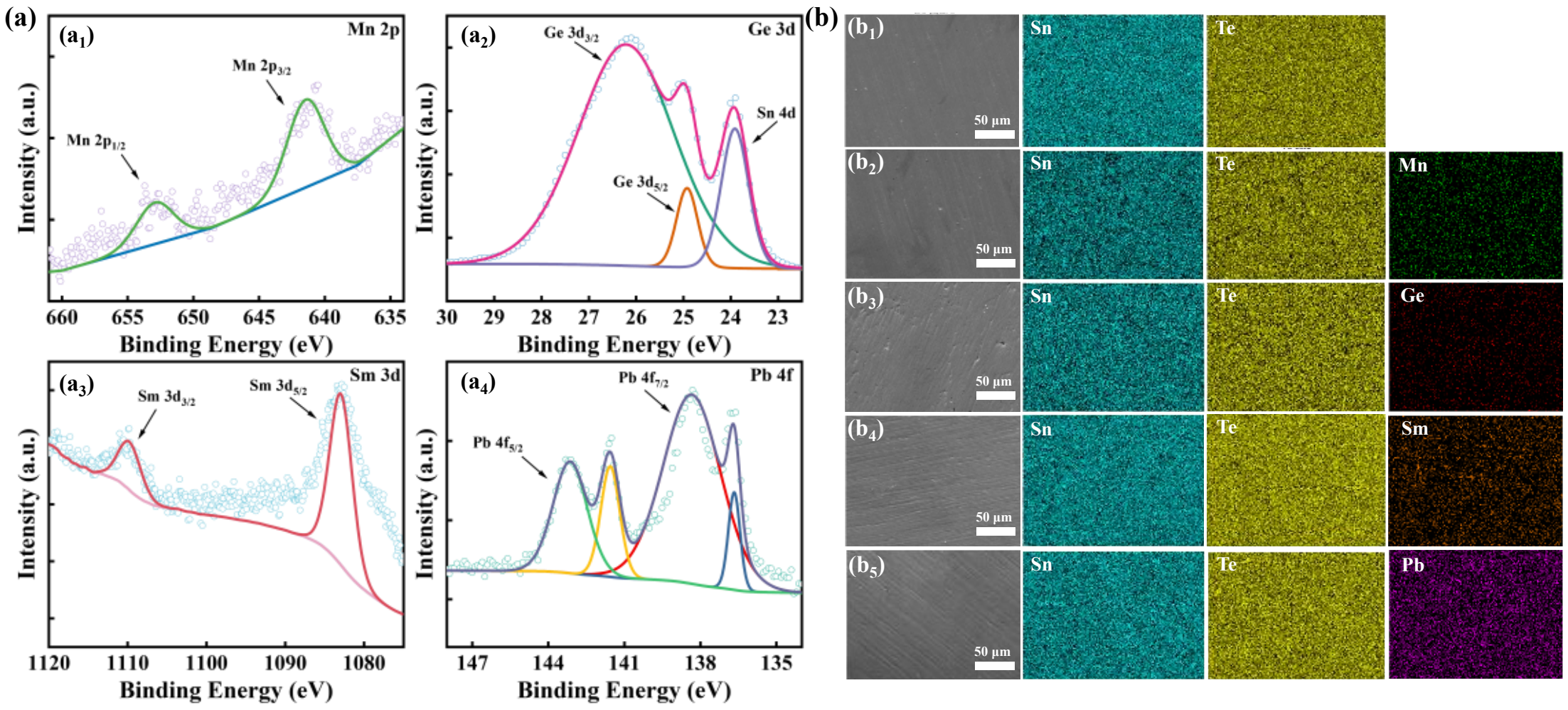

Figure S1. (a) X-ray photoelectron spectroscopy (XPS) results for Mn, Ge, Sm and Pb elements of $Sn_{0.95}M_{0.05}Te$. (b) Scanning electron microscopy (SEM) and energy-dispersive X-ray spectroscopy (EDS) images of $Sn_{0.95}M_{0.05}Te$.

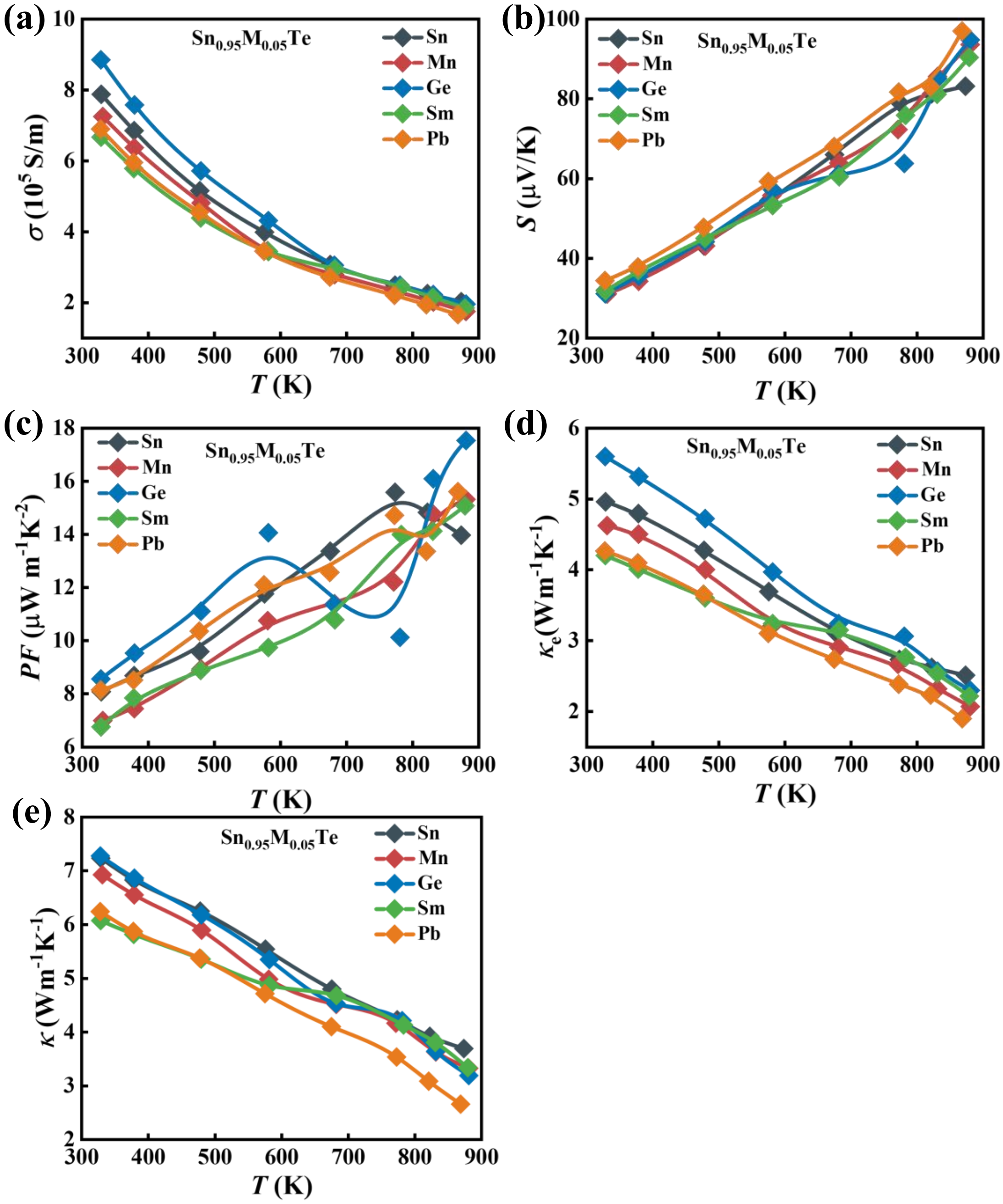

Figure S2. The temperature dependence of (a) electrical conductivity, (b) Seebeck coefficient, (c) power factor (*PF*), (d) electronic thermal conductivity and (e) total thermal conductivity for $Sn_{0.95}M_{0.05}Te$ (M = Sn, Mn, Ge, Sm, and Pb).

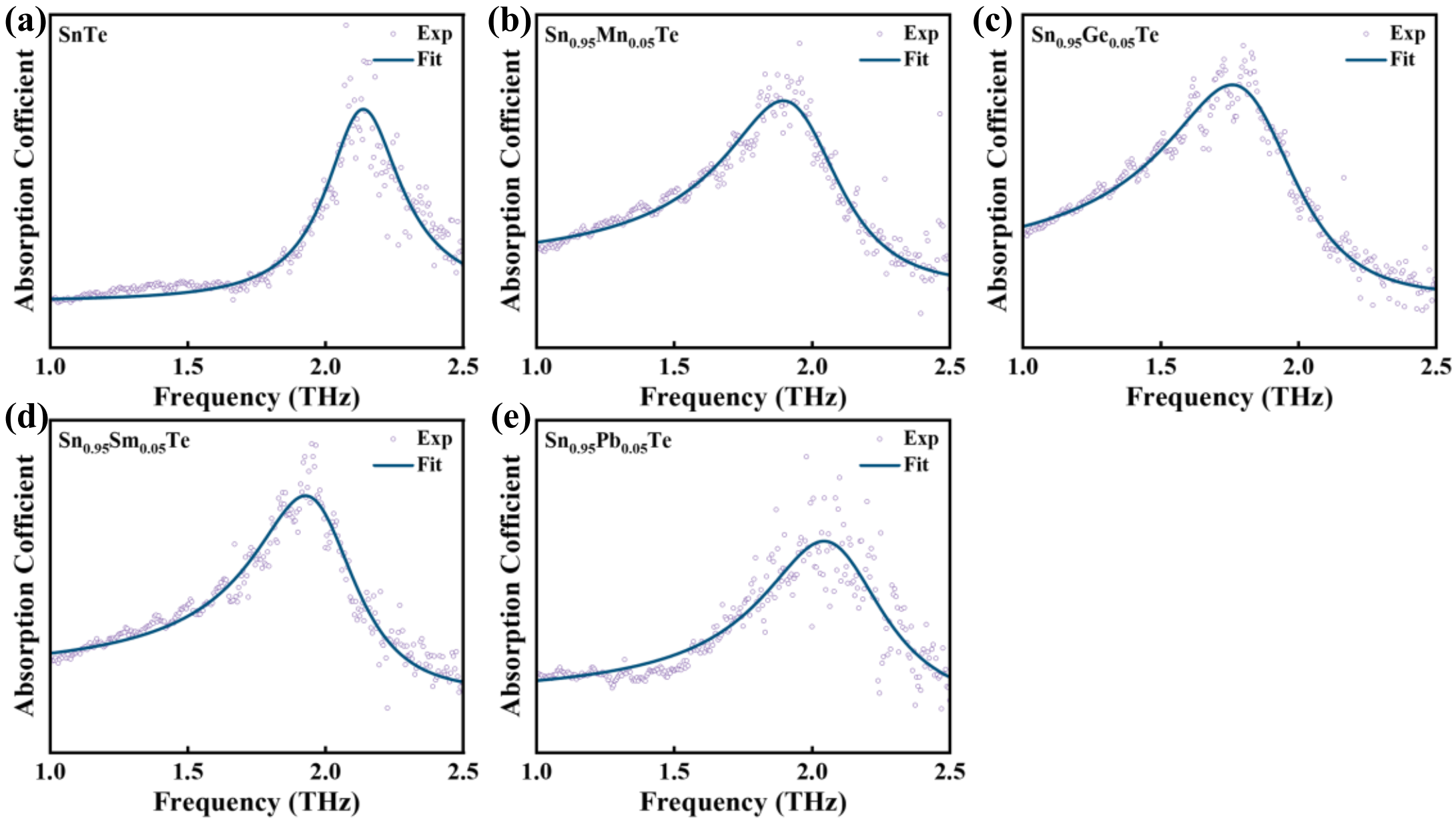

Figure S3. Measured and fitted Γ-point TO phonon peaks in the THz time-domain spectra of $Sn_{0.95}M_{0.05}Te$ (M = Sn, Mn, Ge, Sm, and Pb).

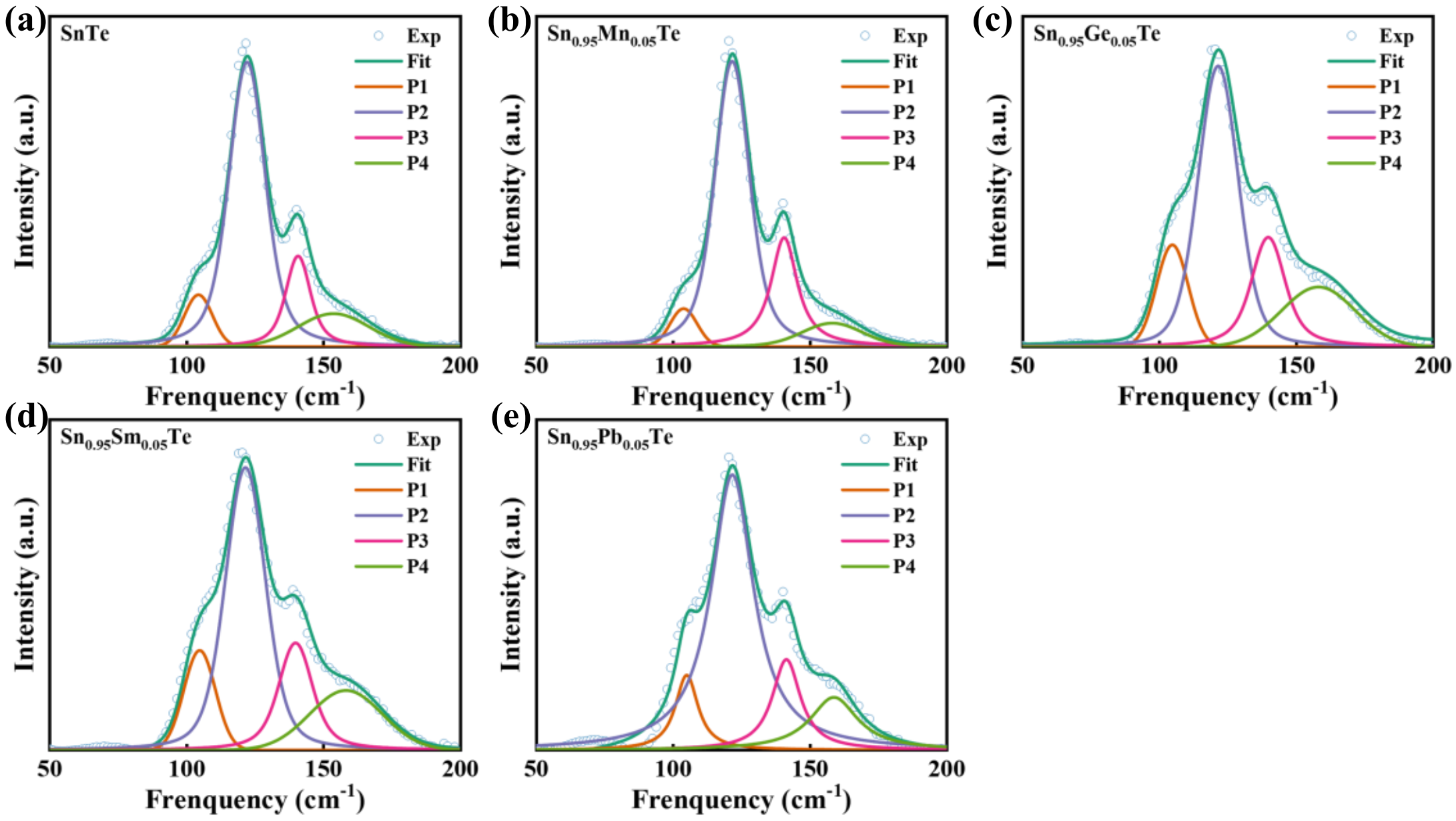

Figure S4. Measured and fitted Raman spectra along with the four peaks P1, P2, P3, and P4 for $Sn_{0.95}M_{0.05}Te$ (M = Sn, Mn, Ge, Sm, and Pb).

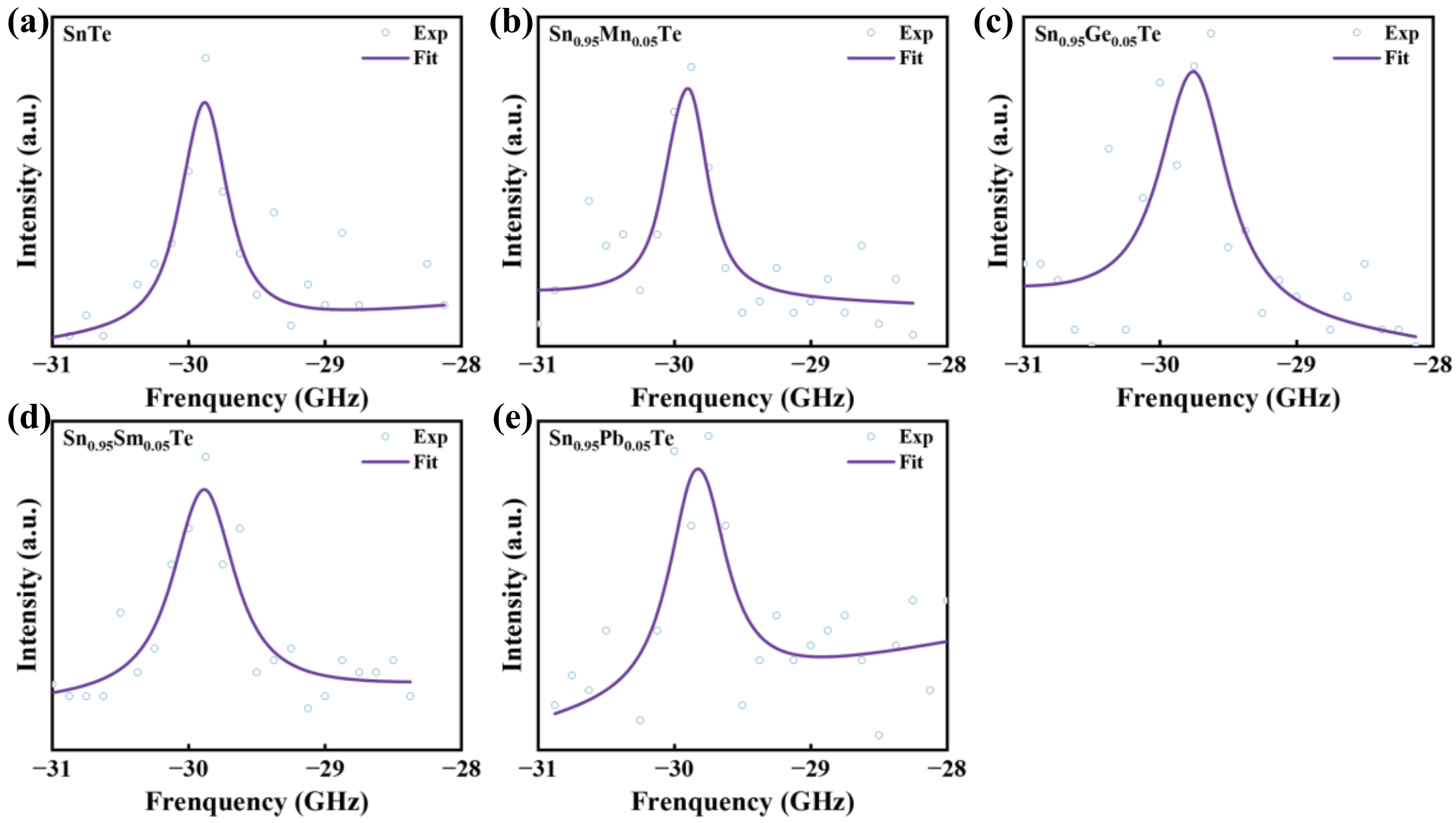

Figure S5. Measured and fitted LA phonon peaks in the Brillouin spectra of $Sn_{0.95}M_{0.05}Te$ (M = Sn, Mn, Ge, Sm, and Pb).

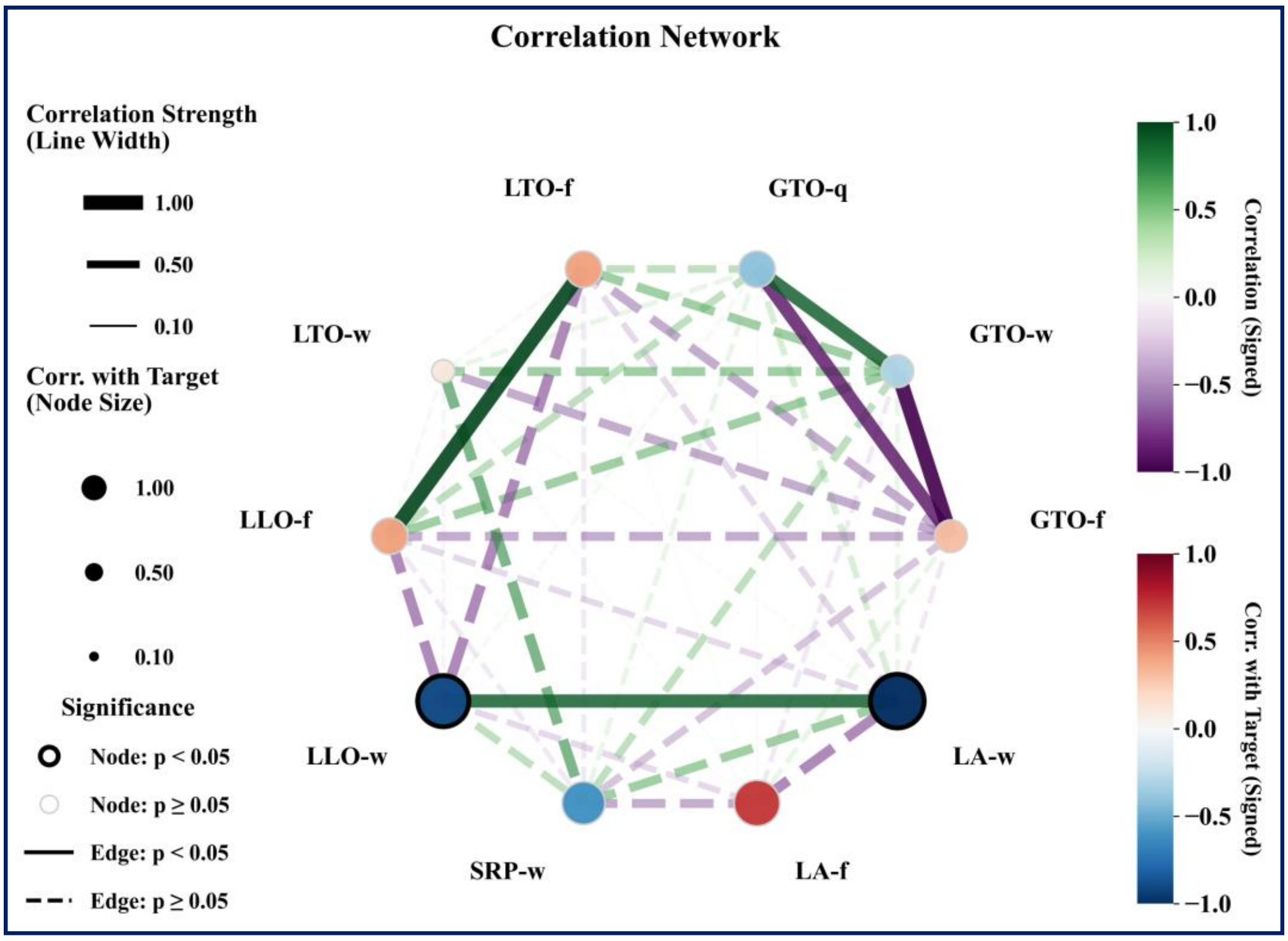

Figure S6. Correlation network diagram with lattice thermal conductivity as the target quantity. The thickness and color of the connecting lines represent the strength of correlation between variables, while solid and dashed lines indicate whether the correlation is statistically significant. The size and color of each node denote the strength of its correlation with the target property, and the presence or absence of a border reflects statistical significance. In the labels, L and G refer to the L and Γ points in the Brillouin zone, respectively, and SRP denotes the second-order Raman process.

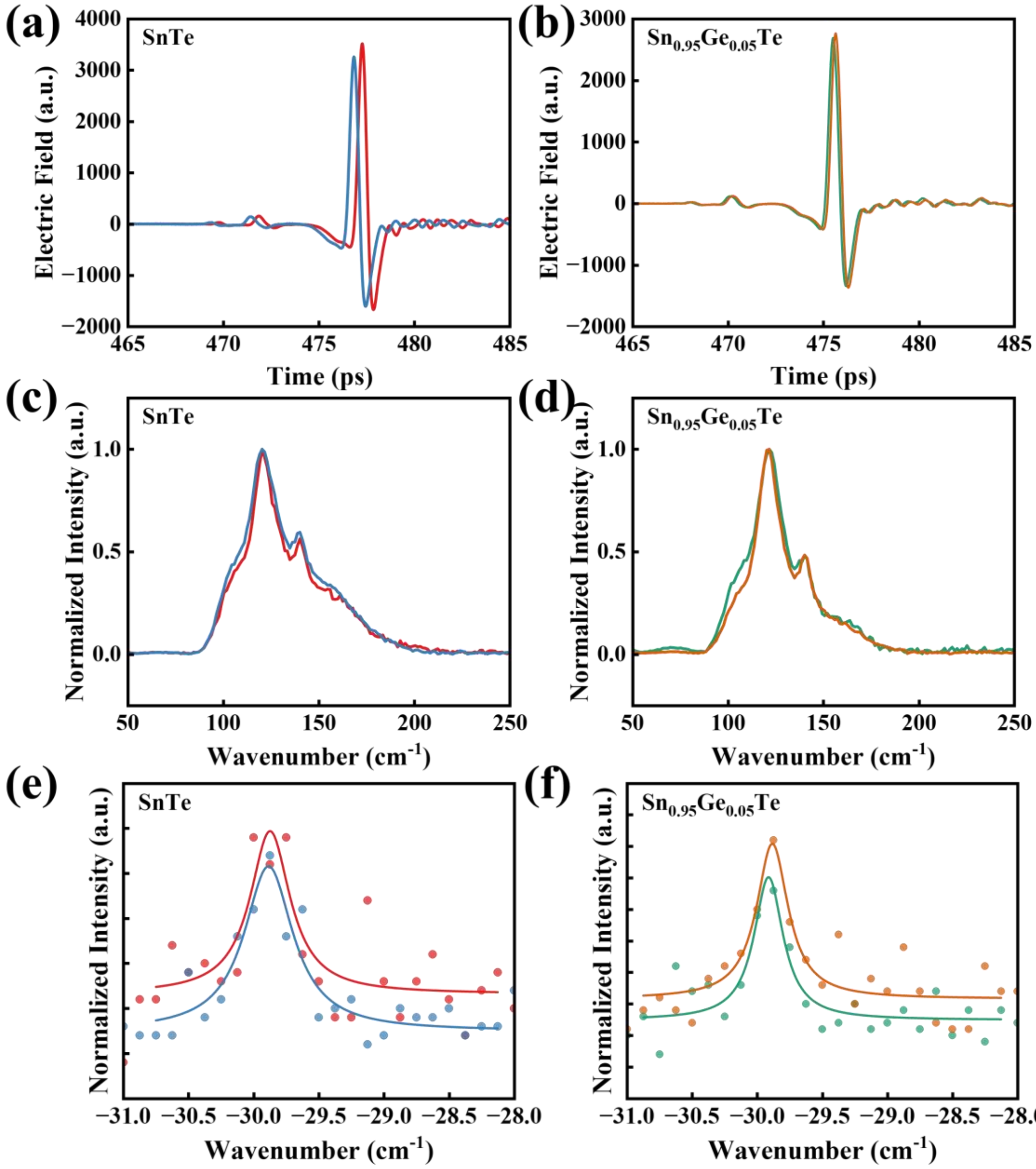

Figure S7. Reproducibility of the spectroscopic measurements for independently synthesized SnTe and $Sn_{0.95}Ge_{0.05}Te$ samples. (a,b) THz, (c,d) Raman, and (e,f) Brillouin spectra obtained from two independently synthesized samples of each composition.

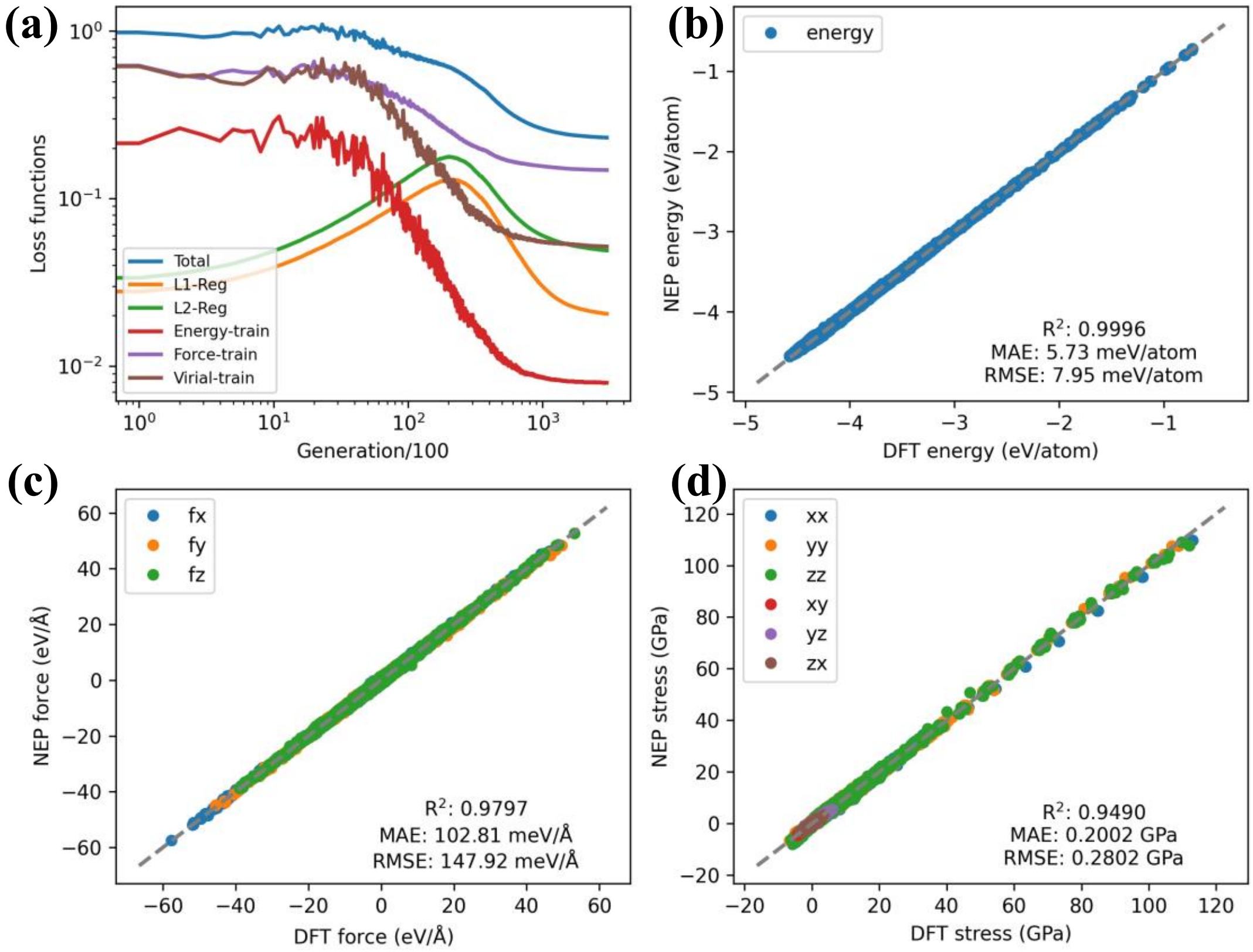

Figure S8. Training process and validation results of the neuroevolution potential (NEP). (a) Evolution of loss functions during training, including total loss (Total), L1 regularization (L1-Reg), L2 regularization (L2-Reg), energy training loss (Energy-train), force training loss (Force-train), and virial training loss (Virial-train). Parity plots comparing NEP predictions against DFT reference values for: (b) atomic energy, (c) atomic forces, and (d) stress tensor components. The gray dashed line denotes the ideal y = x function. The coefficient of determination (R^2), mean absolute error (MAE), and root-mean-square error (RMSE) are indicated in each panel.

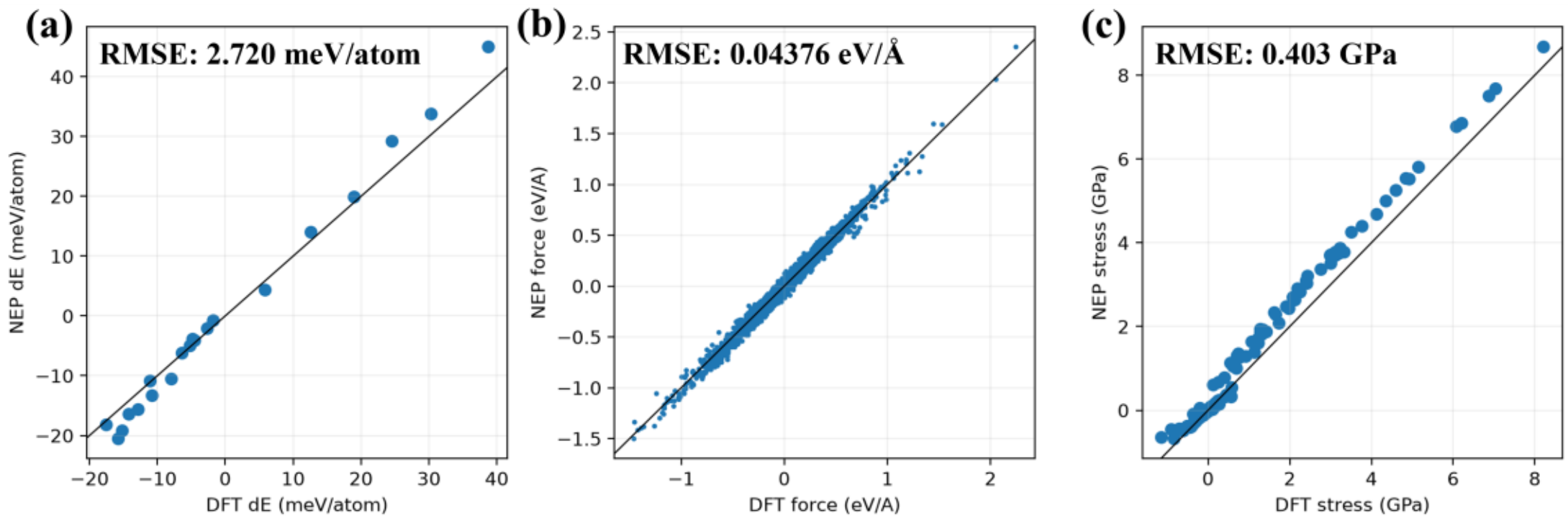

Figure S9. DFT–NEP comparison for an independent test set of randomly distorted 128-atom substituted structures containing three M atoms: (a) relative energies, (b) atomic forces, and (c) stresses. The solid black lines denote perfect agreement.

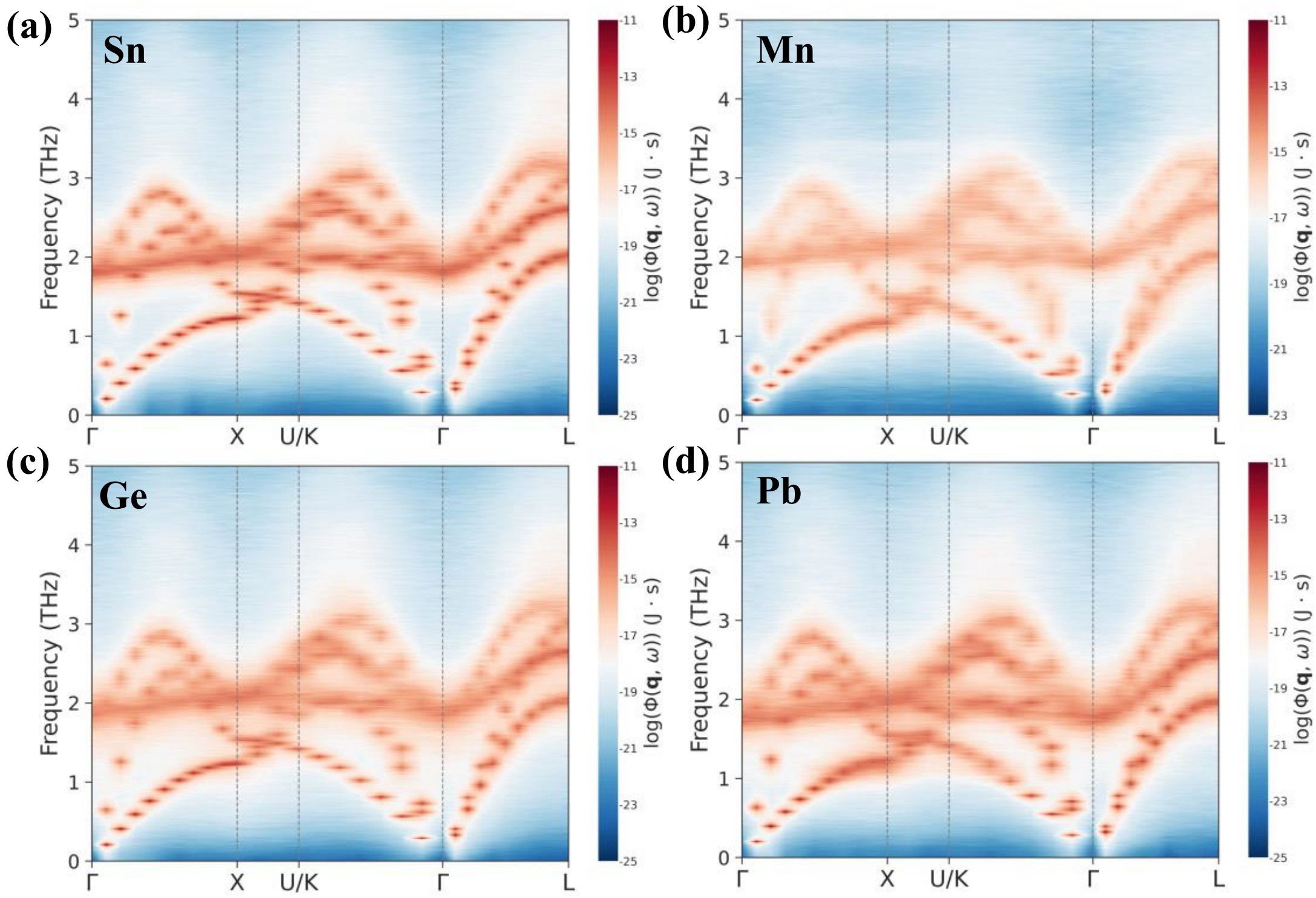

Figure S10. The spectral energy density (SED) of $Sn_{0.95}M_{0.05}Te$ (M = Sn, Mn, Ge, and Pb) from atomic velocities obtained in molecular dynamics (MD) simulations based on NEP potential.

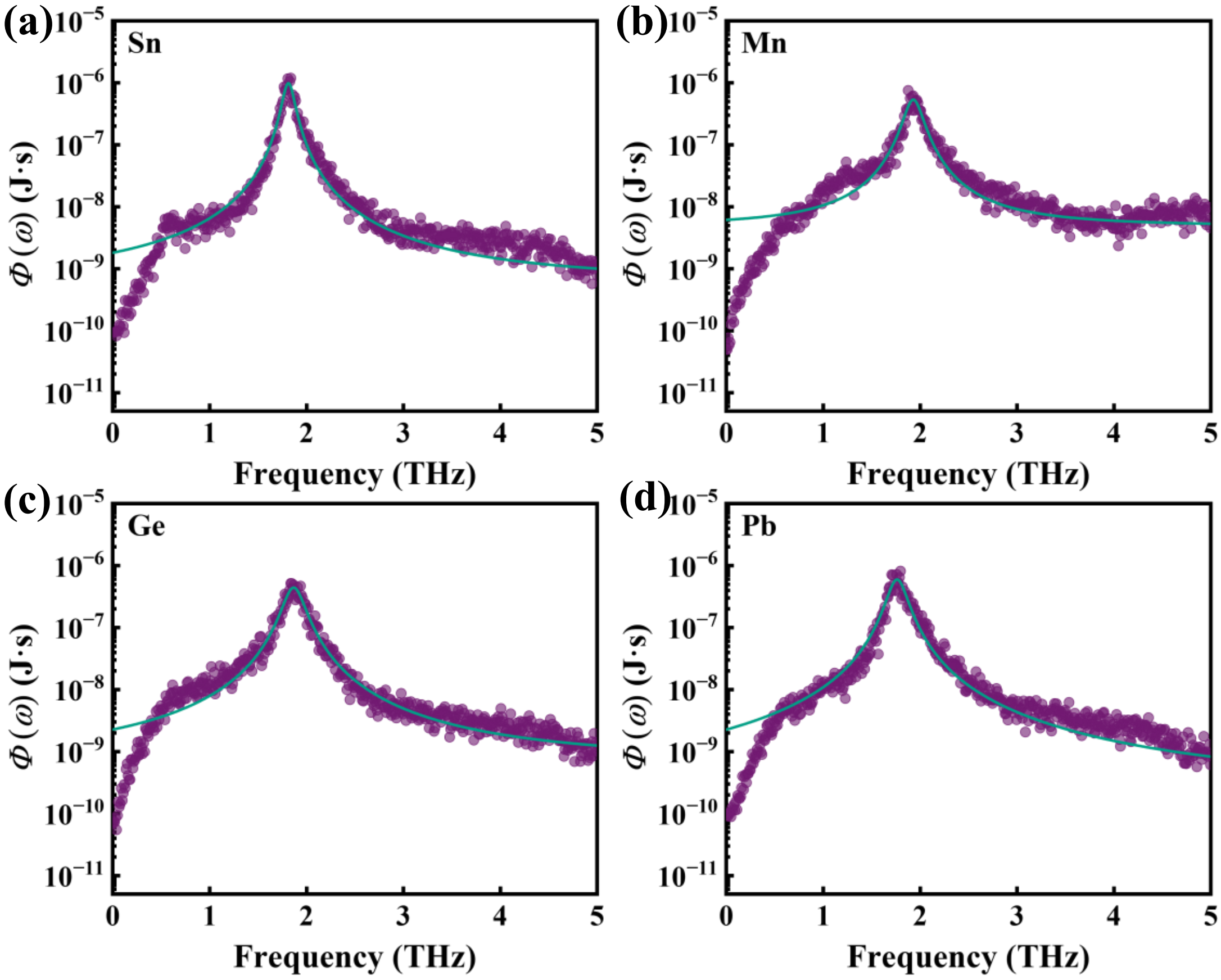

Figure S11. Spectral energy density at the Γ-point for (a) M=Sn, (b) M=Mn, (c) M=Ge, and (d) M=Pb. The purple circles represent the raw SED data obtained from molecular dynamics simulations, and the cyan solid lines show the Lorentzian function fits to the dominant phonon peaks.

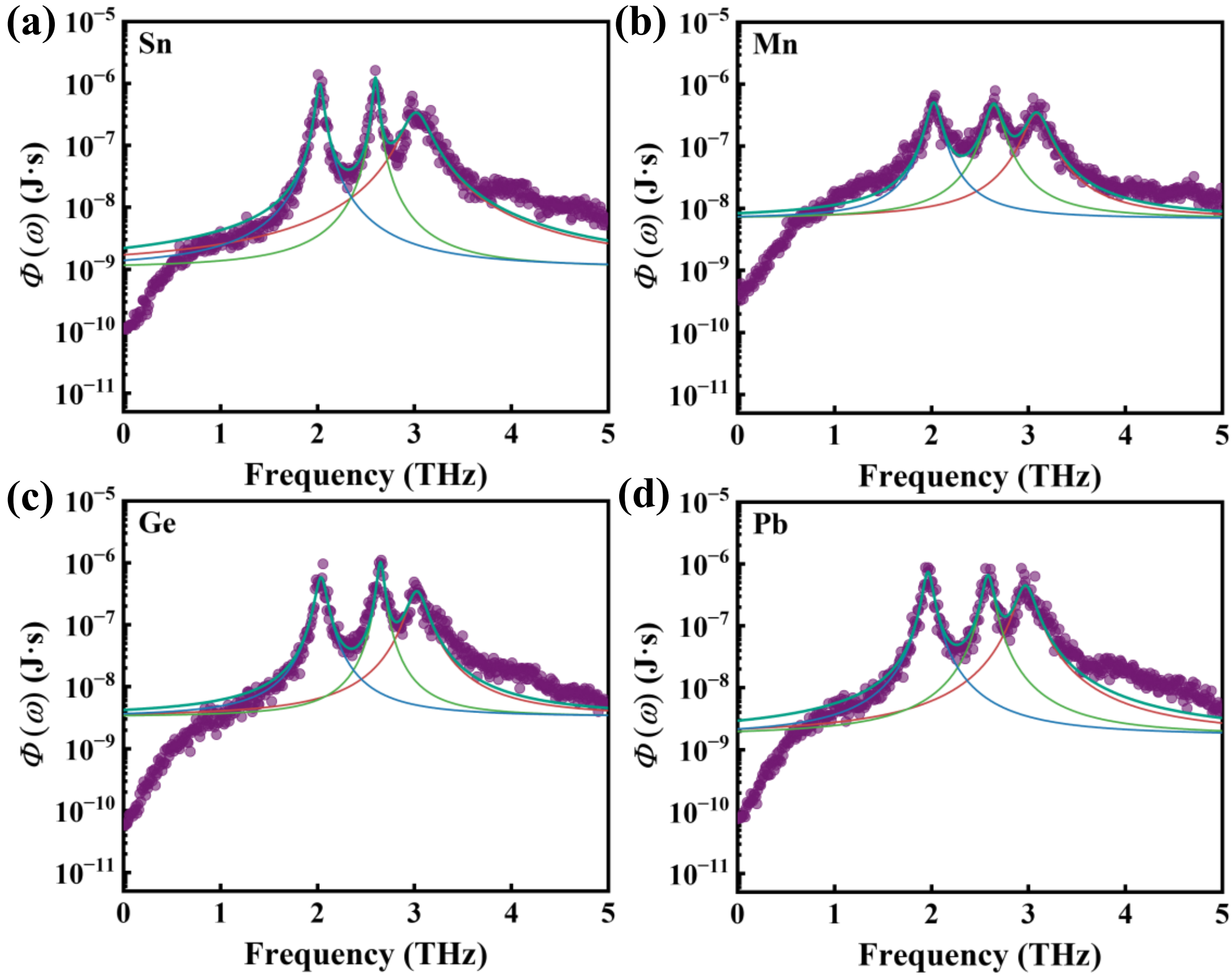

Figure S12. Spectral energy density at the *L*-point for (a) M=Sn, (b) M=Mn, (c) M=Ge, and (d) M=Pb.
